# Dual-domain self-supervised feature alignment via spectral–spatial representation learning for deepfake anomaly detection

**DOI:** 10.1371/journal.pone.0358049

**Published:** 2026-09-11

**Authors:** Yi He

**Affiliations:** 1 School of Computer and Data Science, Henan University of Urban Construction, Pingdingshan, Henan, China; 2 Batangas State University, The National Engineering University, Batangas City, Batangas, Philippines; Dalian Polytechnic University, CHINA

## Abstract

Deepfake anomaly detection has become increasingly critical in visual security applications. However, many existing methods rely heavily on large-scale supervised forgery annotations and often struggle to capture subtle manipulation traces distributed across both spatial and frequency domains. To address these limitations, we propose a Self-Supervised Dual-Domain Alignment (SDDA) framework that jointly learns spatial and spectral representations without requiring labeled forged samples. Specifically, SDDA employs two parameter-sharing encoders to extract complementary features from RGB images and their frequency-domain counterparts, while a cross-domain alignment module enforces consistency between spatial textures and spectral signatures. To provide explicit and reproducible self-supervised supervision, four complementary descriptors are further derived from FAN feature maps: Local Structural Deviation (LSD) for local gradient inconsistency, Global Pattern Discrepancy (GPD) for holistic channel-correlation differences, Local Residual Difference (LRD) for residual-level inconsistency, and Total Consistency Deviation (TCD) for multi-layer feature deviation. These descriptors are predicted from the fused spatial–frequency embedding, encouraging the model to learn manipulation-sensitive representations in the absence of fake samples. In addition, a dual-domain contrastive objective enhances the discrimination of subtle forgery-related anomalies while improving robustness against real-world degradations such as compression and illumination variations. Experimental results demonstrate that SDDA consistently outperforms state-of-the-art baselines, particularly under challenging unseen-manipulation scenarios. Overall, the proposed framework provides an interpretable and label-efficient solution for dual-domain deepfake anomaly detection.

## 1 Introduction

Deepfake anomaly detection has become increasingly crucial in visual security applications, including identity authentication, surveillance, and multimedia forensics [1,2]. The main objective is to identify facial manipulations that deviate from genuine human appearance. However, reliable detection remains challenging due to complex illumination conditions, compression artifacts, and subtle low-level inconsistencies introduced by advanced generative models [3,4].

Early studies primarily relied on supervised deep networks such as Xception-based classifiers [5], capsule networks [6], and attention-guided CNNs [7]. Although these supervised models achieved strong results on known manipulation types, they tend to overfit dataset-specific visual cues, resulting in poor generalization to unseen forgeries [2]. To address this issue, unsupervised and one-class anomaly detection approaches—such as autoencoders [8], DeepSVDD [9], and GANomaly [10]—have been explored to model normal facial appearance and detect deviations as anomalies. Despite their advantages, spatial reconstruction-based frameworks often struggle to capture subtle, high-frequency inconsistencies introduced during synthesis and compression.

**Fig 1:**
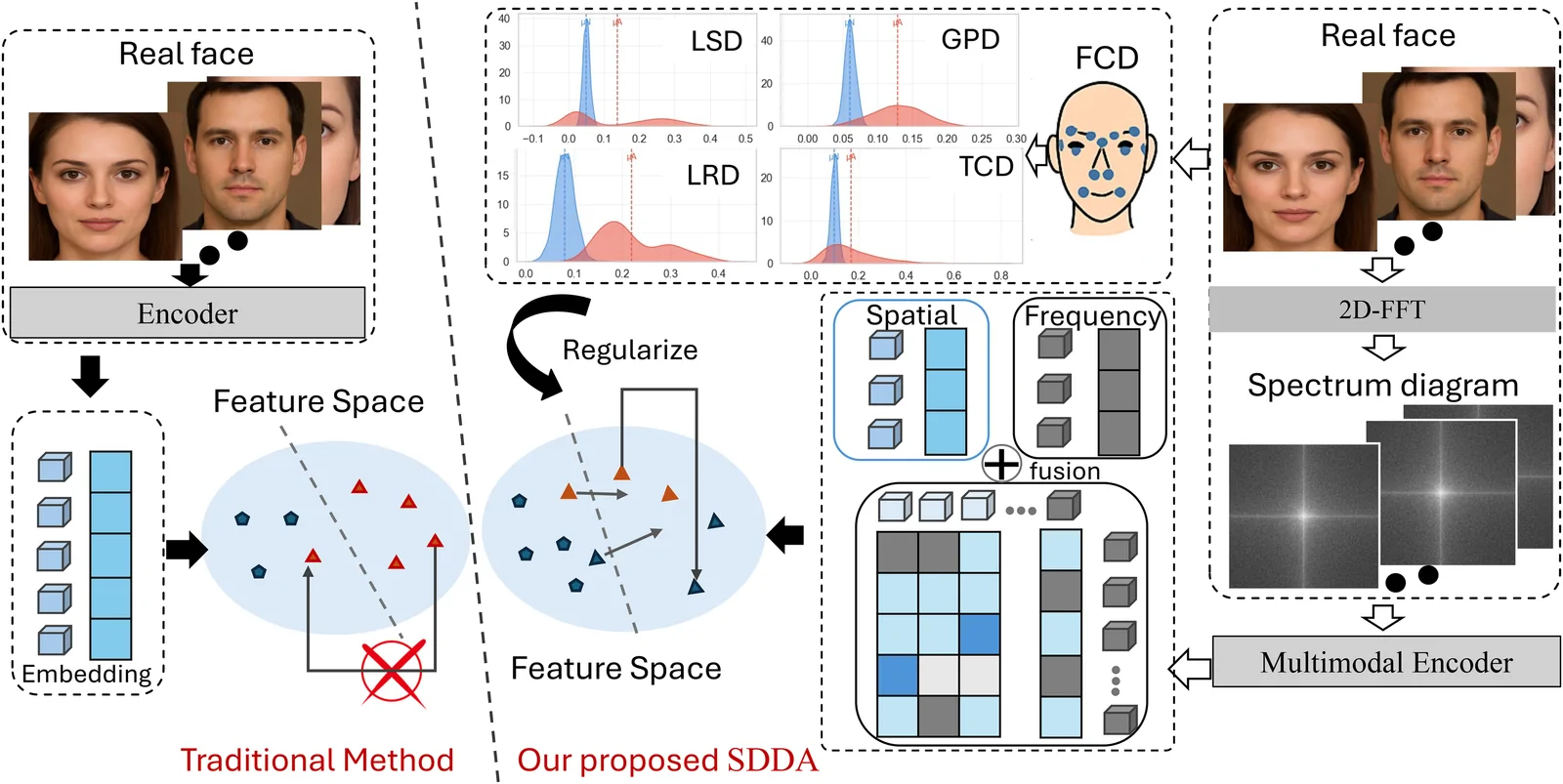
Motivation of the proposed framework. Traditional methods rely solely on spatial reconstruction or supervised classification, failing to capture frequency-domain inconsistencies. Our method introduces a dual-domain self-supervised learning paradigm that jointly models spatial and spectral representations through shared-weight encoders, enhancing robustness and discriminability.

To enhance robustness, frequency-domain cues have recently been incorporated into deepfake and anomaly detection. Frank *et al.* [11] demonstrated that spectral magnitude discrepancies offer discriminative power across different forgery generators. Similarly, dual-domain architectures such as DCT-enhanced discriminators [12] and multi-branch frequency CNNs [13] attempted to fuse spatial and spectral features. Nevertheless, these approaches still depend heavily on supervised labels or handcrafted fusion strategies, limiting adaptability and interpretability [14]. As illustrated in Fig 1, existing deepfake anomaly detection frameworks often rely solely on pixel-level reconstruction or frequency-only artifacts. Such single-domain modeling restricts their ability to learn joint representations that capture both global structural integrity and local spectral inconsistencies. Moreover, reconstruction-based learning tends to produce overly smooth outputs, losing the fine-grained discriminative cues vital for anomaly localization.

To overcome these limitations, we propose a dual-domain self-supervised deepfake anomaly detection framework. Our method employs shared-weight encoders to process both the spatial image and its Fourier spectrum, enabling consistent and aligned cross-domain representation learning. To further enhance discriminability, we introduce a multi-feature prediction task comprising four complementary objectives: Local Structural Deviation (LSD), Global Pattern Discrepancy (GPD), Local Residual Difference (LRD) and Total Consistency Deviation (TCD), as summarized in Table 1. These self-supervised objectives encourage the encoder to capture fine-grained variations at both the global and local levels and to model intrinsic correlations between spatial and frequency domains. Finally, we design a unified anomaly scoring mechanism that combines global reconstruction discrepancies with local frequency-prediction errors, improving robustness and interpretability for open-world detection scenarios.

**Table 1:** Mathematical definitions of the four self-supervised feature objectives in SDDA.

Feature	Mathematical Definition
**LSD** (Local Structural Deviation)	$\ell =\frac{1}{N}\sum_{i=1}^{N}|\nabla I_{s}(i)-\nabla I_{f}(i){|}^{2}$
**GPD** (Global Pattern Discrepancy)	$\gamma =\|\frac{1}{N}\sum_{i=1}^{N}I_{s}(i)-\frac{1}{N}\sum_{i=1}^{N}I_{f}(i){\|}_{2}^{2}$
**LRD** (Local Residual Difference)	$\rho =\frac{1}{N}\sum_{i=1}^{N}|(I_{s}(i)-{\hat{I}}_{s}(i))-(I_{f}(i)-{\hat{I}}_{f}(i))|$
**TCD** (Total Consistency Deviation)	$\tau =\frac{1}{M}\sum_{k=1}^{M}|\phi_{s}^{k}-\phi_{f}^{k}{|}^{2}$
	where $\phi_{s}^{k}$ and $\phi_{f}^{k}$ denote the *k*-th layer features in the spatial and frequency encoders.

We evaluate our framework on five public datasets—FaceForensics++ [5], Celeb-DF [2], Flickr-Faces-HQ [15], WildDeepfake [16], and ForgeryNet [17]. Extensive experiments demonstrate that our method consistently surpasses state-of-the-art baselines, yielding substantial improvements in AUC and average precision (AP). Unlike prior frequency-aware or self-supervised methods operating on single-domain representations, our approach explicitly enforces spatial–frequency alignment via multi-feature prediction, providing both enhanced generalization and greater interpretability in deepfake anomaly detection.

The major contributions of this work are as follows:

- We propose a dual-domain self-supervised framework that jointly models spatial and frequency representations via shared-weight encoders.
- We introduce a multi-feature prediction strategy to estimate four domain-consistent features (ℓ, γ, ρ, τ), strengthening local–global alignment across domains.
- We design a unified anomaly scoring mechanism that integrates global reconstruction errors with local frequency prediction discrepancies for robust and interpretable detection.
- Extensive experiments on four benchmarks confirm the superiority of our method over existing state-of-the-art approaches in both AUC and AP.

## 2 Related works

### 2.1 Traditional methods

Early deepfake detection and general anomaly identification [18] approaches primarily relied on handcrafted features and shallow classifiers to distinguish authentic facial images from manipulated ones. Zhang *et al.* [19] provided a comprehensive review of deepfake audio detection, summarizing forgery mechanisms, detection pipelines, datasets, and evaluation protocols while also discussing emerging challenges related to privacy, robustness, and fairness. Traditional visual forgery detection typically focused on spatial inconsistencies, illumination artifacts, or codec-induced traces. For instance, Li *et al.* [20] exploited physiological cues such as eye blinking and head pose abnormalities, while Matern *et al.* [21] examined texture descriptors and facial color statistics. In the frequency domain, Durall *et al.* [22] demonstrated that upsampling operations in generative models introduce distinctive spectral artifacts that can be used for detection.

To overcome the brittleness of handcrafted features under compression, perturbations, or unseen manipulation types, reconstruction-based anomaly detection frameworks gained popularity. Autoencoders [8], DeepSVDD [9], and GANomaly [10] learn compact representations of normal samples and identify anomalies through reconstruction deviation or embedding distance. Further developments such as AnoGAN [23], DRAEM [24], and PatchCore [25] enhanced robustness using adversarial priors or patch-level reasoning. Nevertheless, purely reconstruction-driven models often fail to capture subtle, high-frequency inconsistencies characteristic of realistic deepfake manipulations, motivating the exploration of frequency-aware and self-supervised strategies.

### 2.2 Deep learning methods

Recent advances in deep learning have significantly improved the ability to capture spatial, temporal, and spectral irregularities in manipulated content. Early CNN-based models such as MesoNet [26] demonstrated strong performance on low-resolution datasets, while RNN-based architectures [27] incorporated temporal dynamics across video frames. Frequency-guided architectures, including F3-Net [12] and FreqFake [22], explicitly modeled Fourier-domain anomalies caused by generative upsampling, improving robustness under cross-manipulation and compression settings. To further enhance generalization ability, Yang *et al.* [28] introduced CSTAN, which integrates multi-dimensional attention and dynamic convolution to better resist previously unseen manipulation types.

More recently, several studies have explored fine-grained spatial–frequency cues and noise-sensitive representations to improve robustness against post-processing and unseen manipulations. Chen *et al.* [29] proposed a signal–noise separation strategy to suppress content-related interference and emphasize manipulation artifacts, while Liao *et al.* [30] leveraged subtle facial muscle motion patterns to enhance deepfake detection under heavy compression. Related investigations in multimedia forensics further indicate that manipulations introduce non-local structural and frequency inconsistencies that are difficult to capture using single-domain representations alone [31,32]. Beyond supervised paradigms, self-supervised and contrastive learning methods have emerged as powerful alternatives for representation learning without relying on labeled fake samples. CLFD [33] leveraged contrastive objectives to learn manipulation-agnostic features, whereas FATNet [34] incorporated frequency-sensitive transformers to emphasize cross-scale spectral inconsistencies. Generative and diffusion-based methods, including DiffusionAD [35] and Reverse Distillation [36], have also shown promise in modeling normality with stronger generative priors. Meanwhile, flow-based approaches such as FastFlow [37], CutPaste [38], and CFlow [39] demonstrated the effectiveness of density estimation and self-distillation for visual anomaly detection. Additional works, such as Walczyna *et al.* [40], examined the destructive effects of deepfake face swapping on digital watermarks, revealing how manipulation propagates non-locally across image regions—further motivating techniques capable of capturing global–local inconsistency. In additional, very recent studies published in 2025 have further explored hybrid spatial–frequency modeling and transformer-based architectures for deepfake detection. Liu *et al.* [41] proposed a frequency-aware vision transformer that explicitly captures long-range spectral dependencies to improve robustness against high-quality forgeries. Similarly, Chen *et al.* [42] introduced a hybrid CNN–Transformer framework that jointly encodes spatial textures and frequency residuals under a supervised learning paradigm. In addition, Stamnas *et al.* [43] proposed DiffFake, which transforms the detection task into “differential anomaly detection.” First, they trained the EfficientNet-b4 backbone with self-mixed pseudo Deepfake (simultaneously implanting global and local artifacts) to make the model sensitive to forgery traces. Then, they extracted 1792-d face embeddings and performed (A–B)² fusion using only real image pairs with the same identity, and learned the “natural variation” distribution using a three-component Gaussian mixture model.

Despite these advances, most existing deepfake detection approaches require large-scale labeled fake datasets or lack mechanisms to jointly model spatial and spectral consistencies. In contrast, our proposed dual-domain self-supervised framework addresses these limitations by integrating shared-weight spatial–frequency encoding, multi-feature prediction, and unified global–local anomaly scoring. This design enables the model to effectively capture both structural context and high-frequency artifacts, providing strong detection performance and superior cross-dataset generalization without explicit fake supervision.

### 2.3 Anomaly detection-based deepfake detection methods

Anomaly detection has become an important and rapidly developing research direction in the field of deepfakes in recent years, especially in addressing the problem of insufficient generalization ability of traditional supervised classification methods in open set scenarios, which has shown significant advantages.

Reconstruction-based methods train reconstruction models (such as autoencoders) using only real faces, allowing them to learn the distribution of real data. During testing, real images are typically reconstructed accurately, while forged images, due to containing aberrant tampering traces, often produce significant reconstruction errors. Deep forgery detection can be achieved by analyzing the differences between the original and reconstructed images. These methods do not require a large number of forged samples and have good generalization ability against unknown forgery methods. Shi et al. [44] proposed FRG2D, an end-to-end face reconstruction-based framework for generalized deepfake detection. The method learns the distribution of authentic faces through reconstruction and identifies manipulated images by analyzing reconstruction discrepancies. To enhance feature extraction, FRG2D incorporates CBAM, CAB, and a Residual Outlook Attention (ROA) module. Experimental results demonstrate that FRG2D achieves strong generalization performance across unseen deepfake domains.

One-class classification methods are trained exclusively on authentic facial images to learn the feature distribution or decision boundary of real data. During inference, samples that deviate from this learned distribution are regarded as anomalies and are therefore classified as deepfakes. Since these methods do not rely on manipulated samples for supervised training, they generally exhibit strong generalization ability to unseen forgery methods. Khalid et al. [45] proposed OC-FakeDect, a one-class deepfake detection method that trains a Variational Autoencoder (VAE) solely on authentic facial images and identifies manipulated images as anomalies, enabling effective generalization to unseen forgery methods. Cao et al. [46] proposed OC-SAN, a one-class style autoencoder network for deepfake detection. The model learns identity and personalized style representations from authentic facial images and reconstructs only real faces effectively. Manipulated images are identified based on abnormal reconstruction patterns, enabling strong generalization to unseen forgery methods without requiring fake samples during training.

Feature distribution modeling methods learn the statistical distribution of authentic facial features and classify samples that deviate significantly from this distribution as deepfakes. By modeling the global structure of real facial representations, these methods generally exhibit strong generalization to unseen forgery methods. Khodabakhsh et al. [47] proposed a two-stage anomaly detection method that learns the distribution of pristine facial images and detects manipulated images based on deviations from the estimated pixel-wise probabilities, achieving strong generalization to unseen synthesis methods. Maiano et al. [48] proposed two out-of-distribution (OOD) detection methods for generalized deepfake detection. The first method detects manipulated images through reconstruction errors, while the second incorporates an attention mechanism to enhance anomaly localization. Both methods are designed to identify samples that deviate from the distribution of authentic images, demonstrating strong robustness to unseen deepfake generation methods.

## 3 Methodology

**Fig 2:**
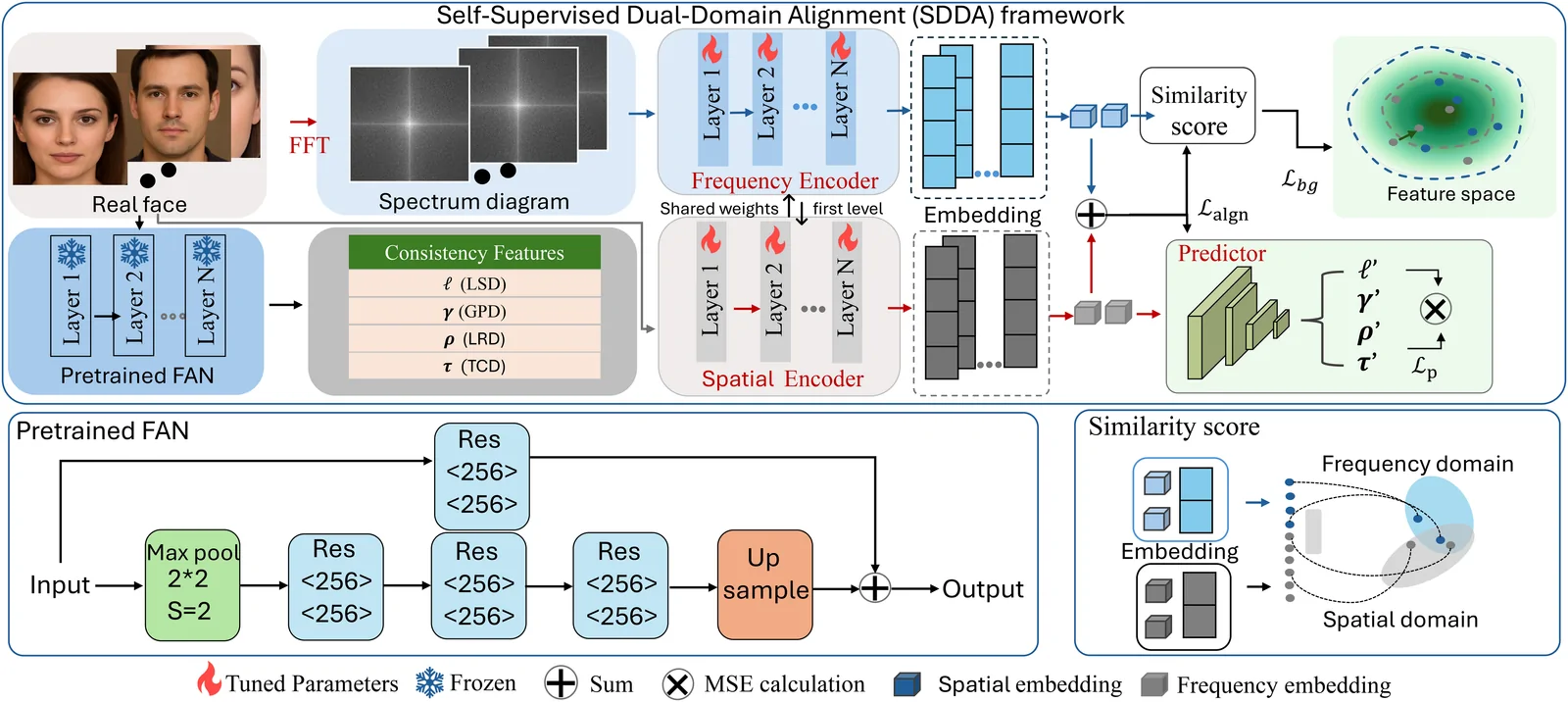
Overview of the proposed Self-Supervised Dual-Domain Alignment (SDDA) framework for deepfake anomaly detection.

In this section, we introduce the proposed SDDA (Dual-domain Feature Alignment for Deepfake Anomaly Detection) framework. The SDDA model is designed as a self-supervised anomaly detection system that jointly learns spatial-frequency consistency and multi-feature prediction, without requiring fake data during training. The overall framework is depicted in Fig 2. SDDA consists of three key components: (1) a dual-domain encoder with shared weights for consistent representation learning, (2) a multi-feature prediction head for structural and frequency-based feature regression, and (3) a unified loss function that integrates feature reconstruction, domain consistency, and hypersphere compactness. The architecture contains three core components: (1) a shared-weight spatial–frequency encoder that learns domain-aligned representations through joint feature extraction; (2) a multi-objective feature-prediction head that estimates the four consistency descriptors (ℓ, γ, ρ, τ), enforcing fine-grained spatial–frequency correspondence and enhancing cross-domain discriminability; (3) a global–local anomaly scoring module that integrates multi-scale reconstruction with attention-guided deviation estimation to capture both structural and spectral abnormalities. By explicitly aligning dual-domain embeddings and constraining their consistency through self-supervision, the framework establishes a theoretically grounded representation space that generalizes effectively to unseen manipulation types.

### 3.1 Dual-domain encoder with shared weights

In this section, we present the proposed Self-Supervised Dual-Domain Alignment (SDDA) framework for deepfake anomaly detection. Unlike conventional supervised methods that rely on large quantities of forged samples, SDDA is trained exclusively on real facial images and aims to learn domain-invariant, frequency-aware representations through a combination of spatial–spectral feature coupling, multi-objective self-supervised prediction, and hypersphere regularization. The complete pipeline is shown in Fig 2.

SDDA consists of four tightly coupled modules:

- **A data preprocessing module** that extracts semantic features from a pretrained FAN network and computes the corresponding frequency-domain representation.
- **A dual-domain shared-weight encoder** that jointly encodes spatial and spectral representations.
- **A multi-feature prediction head** that enforces four complementary self-supervised constraints.
- **A multi-objective optimization mechanism** integrating feature prediction, domain alignment, and hypersphere compactness for anomaly scoring.

### 3.2 Data preprocessing: Semantic feature extraction and spectral transformation

Throughout this paper, we denote the input face image in the spatial (RGB) domain as ${𝐗}_{s}\in {\mathbb{R}}^{H\times W\times 3}$. Its corresponding frequency-domain representation, obtained via a two-dimensional discrete Fourier transform (DFT), is denoted as ${𝐗}_{f}$. Unless otherwise specified, ${𝐗}_{s}$ and ${𝐗}_{f}$ always refer to raw spatial images and their frequency spectra, respectively. Feature maps extracted by the pretrained FAN network are explicitly denoted as ${𝐅}_{s}$ and ${𝐅}_{f}$ to avoid ambiguity.

We adopt the fast Fourier transform (FFT) as the frequency-domain transformation for two primary reasons. First, FFT explicitly reveals periodic patterns and high-frequency artifacts introduced by common deepfake generation operations, such as upsampling, blending, and post-processing compression, which are difficult to capture purely in the spatial domain. Second, FFT provides a deterministic and parameter-free spectral decomposition, avoiding additional learnable parameters that may bias the normality modeling process in self-supervised anomaly detection. Compared to alternative frequency representations such as wavelet transforms or learnable spectral filters, FFT offers superior computational efficiency and stable frequency responses, making it well-suited for large-scale training and cross-dataset generalization. This design choice allows SDDA to effectively leverage spatial–spectral consistency while maintaining robustness to unseen manipulation patterns.

### 3.3 Dual-domain shared-weight encoder

The dual-domain encoder aims to learn a *domain-invariant representation* that aligns spatial and frequency semantics. A single encoder $E_{\theta }(\cdot )$ with shared parameters is applied to both domains:


$$\begin{array}{c} {𝐳}_{s}=E_{\theta }({𝐗}_{s}),\;{𝐳}_{f}=E_{\theta }({𝐗}_{f}), \end{array}$$ (1)

where ${𝐳}_{s},{𝐳}_{f}\in {\mathbb{R}}^{d}$ denote spatial and frequency embeddings.

Weight sharing forces the encoder to capture cross-domain semantic invariances and suppress domain-specific artifacts. This improves the robustness and generalization of the learned embedding space, especially under unseen manipulation types.

### 3.4 Self-supervised descriptor prediction module

The four self-supervised descriptors are originally defined on the paired spatial and frequency representations $I_{s}$ and $I_{f}$, as summarized in Table 1. To keep the formulation consistent with the original image-level definition, we do not redefine these descriptors at the feature level. Instead, we instantiate $I_{s}$ and $I_{f}$ with the response maps extracted from different layers of the two encoders during implementation.

Given the feature maps ${𝐅}_{s}^{\left(\ell \right)}$ and ${𝐅}_{f}^{\left(\ell \right)}$ from the ℓ-th layer of the spatial and frequency encoders, we first obtain their channel-averaged response maps:


$$\begin{array}{c} I_{s}^{\left(\ell \right)}=\frac{1}{C_{\ell }}\sum_{c=1}^{C_{\ell }}{𝐅}_{s,c}^{\left(\ell \right)},\;I_{f}^{\left(\ell \right)}=\frac{1}{C_{\ell }}\sum_{c=1}^{C_{\ell }}{𝐅}_{f,c}^{\left(\ell \right)}. \end{array}$$ (2)

Here, $I_{s}^{\left(\ell \right)}$ and $I_{f}^{\left(\ell \right)}$ are regarded as layer-wise instantiations of the original spatial and frequency representations $I_{s}$ and $I_{f}$. Therefore, the following descriptors preserve the same mathematical meaning as the image-level definitions, while allowing them to be computed from the selected encoder layers.

**Local Structural Deviation.** Local Structural Deviation (LSD) measures local structural inconsistency between the spatial and frequency representations. Following the original definition, LSD is computed as the average gradient discrepancy:


$$\begin{array}{c} y_{LSD}=\frac{1}{N_{2}}\sum_{i=1}^{N_{2}}{\left|\nabla I_{s}^{\left(2\right)}(i)-\nabla I_{f}^{\left(2\right)}(i)\right|}^{2}, \end{array}$$ (3)

where $I_{s}^{\left(2\right)}$ and $I_{f}^{\left(2\right)}$ denote the response maps extracted from the second encoder layer, and *N*_2_ is the number of spatial positions in the response map. The gradient operator ∇ is implemented by local horizontal and vertical gradient filters. This descriptor encourages the model to preserve fine-grained local structural differences between the two domains.

**Global Pattern Discrepancy.** Global Pattern Discrepancy (GPD) captures the global distribution difference between the spatial and frequency representations. Consistent with the original image-level formulation, GPD is computed as the discrepancy between the mean responses of the two domains:


$$\begin{array}{c} y_{GPD}={\left\|\frac{1}{N_{3}}\sum_{i=1}^{N_{3}}I_{s}^{\left(3\right)}(i)-\frac{1}{N_{3}}\sum_{i=1}^{N_{3}}I_{f}^{\left(3\right)}(i)\right\|}_{2}^{2}, \end{array}$$ (4)

where $I_{s}^{\left(3\right)}$ and $I_{f}^{\left(3\right)}$ are obtained from the third encoder layer. Compared with LSD, GPD focuses more on holistic response distribution and global cross-domain consistency.

**Local Residual Difference.** Local Residual Difference (LRD) measures residual-level inconsistency between the spatial and frequency representations. Following the original definition, we first obtain the locally smoothed responses:


$$\begin{array}{c} {\hat{I}}_{s}^{\left(3\right)}={\mathrm{AvgPool}}_{r}(I_{s}^{\left(3\right)}),\;{\hat{I}}_{f}^{\left(3\right)}={\mathrm{AvgPool}}_{r}(I_{f}^{\left(3\right)}), \end{array}$$ (5)

where ${\mathrm{AvgPool}}_{r}(\cdot )$ denotes average pooling with an r×r kernel. The LRD descriptor is then computed as:


$$\begin{array}{c} y_{LRD}=\frac{1}{N_{3}}\sum_{i=1}^{N_{3}}\left|\left(I_{s}^{\left(3\right)}(i)-{\hat{I}}_{s}^{\left(3\right)}(i)\right)-\left(I_{f}^{\left(3\right)}(i)-{\hat{I}}_{f}^{\left(3\right)}(i)\right)\right|. \end{array}$$ (6)

This descriptor emphasizes local residual differences and is useful for capturing subtle manipulation traces that may be weakened in global representations.

**Total Consistency Deviation.** Total Consistency Deviation (TCD) measures the consistency deviation between deeper spatial and frequency representations. In accordance with the original definition, TCD is computed over multiple encoder layers. In our implementation, we use the second, third and fourth layers, denoted as ℒ={2,3,4}. For each layer, the feature representation is obtained by global average pooling:


$$\begin{array}{c} \phi_{s}^{\left(\ell \right)}=\mathrm{GAP}({𝐅}_{s}^{\left(\ell \right)}),\;\phi_{f}^{\left(\ell \right)}=\mathrm{GAP}({𝐅}_{f}^{\left(\ell \right)}). \end{array}$$ (7)

The TCD descriptor is formulated as:


$$\begin{array}{c} y_{TCD}=\frac{1}{\left|ℒ\right|}\sum_{\ell \in ℒ}\frac{{\left\|\phi_{s}^{\left(\ell \right)}-\phi_{f}^{\left(\ell \right)}\right\|}_{2}^{2}}{C_{\ell }}. \end{array}$$ (8)

This descriptor provides a multi-level consistency constraint by aggregating deviations from different semantic layers.

The four descriptors are then concatenated to form the self-supervised target vector:


$$\begin{array}{c} 𝐲=[y_{LSD},y_{GPD},y_{LRD},y_{TCD}]\in {\mathbb{R}}^{4}. \end{array}$$ (9)

A prediction head $P_{\phi }(\cdot )$ takes the concatenated spatial–frequency embedding as input:


$$\begin{array}{c} 𝐳=[{𝐳}_{s}\|{𝐳}_{f}], \end{array}$$ (10)

and predicts the descriptor vector:


$$\begin{array}{c} \hat{𝐲}=P_{\phi }(𝐳). \end{array}$$ (11)

The descriptor prediction loss is formulated as:


$$\begin{array}{c} {ℒ}_{p}=\frac{1}{N}\sum_{i=1}^{N}\sum_{m\in ℳ}\lambda_{m}{\left\|{\hat{y}}_{m}^{\left(i\right)}-y_{m}^{\left(i\right)}\right\|}_{2}^{2}, \end{array}$$ (12)

where ℳ={LSD,GPD,LRD,TCD}, and $\lambda_{m}$ denotes the loss weight of each descriptor. In our implementation, all $\lambda_{m}$ are set to 1 unless otherwise specified. To avoid scale imbalance among different descriptors, each descriptor is normalized before loss computation.

### 3.5 Domain alignment loss

To explicitly enforce cross-domain consistency, we introduce an alignment loss defined as the squared Euclidean distance between spatial and frequency embeddings:


$$\begin{array}{c} {ℒ}_{\text{algn}}=\frac{1}{N}\sum_{i=1}^{N}{\left\|z_{s}^{\left(i\right)}-z_{f}^{\left(i\right)}\right\|}_{2}^{2}. \end{array}$$ (13)

This objective encourages the shared-weight encoder to learn domain-invariant representations across spatial and spectral inputs.

### 3.6 Hypersphere compactness constraint

Inspired by DeepSVDD [9], SDDA regularizes normal samples into a compact hypersphere. The hypersphere center is defined as:


$$\begin{array}{c} 𝐜=\frac{1}{N}\sum_{i=1}^{N}{𝐳}_{s}^{\left(i\right)}. \end{array}$$ (14)

The compactness constraint is:


$$\begin{array}{c} {ℒ}_{svdd}=\frac{1}{N}\sum_{i=1}^{N}\left(\|{𝐳}_{s}^{\left(i\right)}-𝐜{\|}_{2}^{2}+\|{𝐳}_{f}^{\left(i\right)}-𝐜{\|}_{2}^{2}\right). \end{array}$$ (15)

This hypersphere formulation increases separation between normal and anomalous embeddings in the joint latent space.

### 3.7 Total optimization objective

SDDA combines the three complementary objectives into a unified loss:


$$\begin{array}{c} {ℒ}_{SDDA}=\lambda {ℒ}_{p}+(1-\lambda )\left({ℒ}_{algn}+{ℒ}_{svdd}\right), \end{array}$$ (16)

where λ controls the trade-off between self-supervised prediction accuracy and structural embedding consistency.

### 3.8 Anomaly scoring

The final anomaly score integrates both global hypersphere deviation and local self-supervised feature prediction error:


$$\begin{array}{c} S(x)=\|𝐳-𝐜{\|}_{2}^{2}+\beta \cdot \|\hat{𝐲}-𝐲{\|}_{2}^{2}, \end{array}$$ (17)

where 𝐳 denotes the latent embedding, 𝐜 is the hypersphere center, and $\hat{𝐲}$ and 𝐲 are the predicted and target self-supervised features, respectively.

The SDDA training procedure is summarized in Algorithm 2.

### 3.9 Design rationale and key innovations scoring

To clarify the novelty of our framework, SDDA is not a simple integration of existing components. Instead, it establishes a unified dual-domain self-supervised learning paradigm, where spatial and frequency representations are explicitly aligned through a consistency constraint rather than directly fused. In addition, a dual-domain contrastive objective is designed to enforce compactness of real samples while implicitly separating manipulated ones as out-of-distribution deviations. Finally, a hypersphere constraint is applied on the aligned representation space to further enhance intra-class compactness and inter-class separability. These designs jointly enable SDDA to effectively capture subtle forgery traces across both domains.

## 4 Experiments

In this section, we describe the datasets, evaluation metrics, baseline methods, and implementation details used in our study. We then comprehensively evaluate the performance of the proposed SDDA framework across multiple public Deepfake anomaly detection benchmarks. Furthermore, ablation studies are conducted to analyze the contribution of each module, including the dual-domain encoder, feature prediction head, and unified loss components. Finally, qualitative visualizations and statistical analyses are provided to demonstrate the interpretability and practical effectiveness of SDDA in detecting subtle and unseen Deepfake manipulations. The general training process of our proposed SDDA model is shown in Table 2.

**Table 2:** Training Procedure of the Proposed SDDA Framework.

Input:
Spatial images $\left\{{𝐗}_{s}\right\}$; frequency images $\left\{{𝐗}_{f}\right\}$;
Pretrained feature targets {𝐲} (FAN features);
Encoder $E_{\theta }$, predictor $P_{\phi }$, hypersphere center 𝐜;
Learning rate η, batch size *B*, total epochs *T*.
**Output:** Trained encoder $E_{\theta }$ and predictor $P_{\phi }$.
**Initialization:**
Initialize parameters θ, ϕ; set optimizer (e.g., Adam) with LR η
**Training Loop:**
**For** *t* = 1 **to** *T*:
1. Shuffle the training set
2. **For** each mini-batch $\left({𝐗}_{s},{𝐗}_{f},𝐲\right)$ of size *B*:
**Forward pass:**
${𝐳}_{s}=E_{\theta }({𝐗}_{s})$ (Spatial embedding)
${𝐳}_{f}=E_{\theta }({𝐗}_{f})$ (Frequency embedding)
$𝐳=[{𝐳}_{s}\|{𝐳}_{f}]$ (Cross-domain joint feature)
$\hat{𝐲}=P_{\phi }(𝐳)$ (Predicted multi-feature vector)
**Loss computation:**
${ℒ}_{p}=\frac{1}{B}\sum_{i=1}^{B}\|{\hat{𝐲}}_{i}-{𝐲}_{i}{\|}_{2}^{2}$ (Prediction loss)
${ℒ}_{algn}=\frac{1}{B}\sum_{i=1}^{B}\|{𝐳}_{s,i}-{𝐳}_{f,i}{\|}_{2}^{2}$ (Domain alignment)
${ℒ}_{bg}=\frac{1}{B}\sum_{i=1}^{B}\left(\|{𝐳}_{s,i}-𝐜{\|}_{2}^{2}+\|{𝐳}_{f,i}-𝐜{\|}_{2}^{2}\right)$ (Hypersphere compactness)
${ℒ}_{SDDA}=\lambda \;{ℒ}_{p}+(1-\lambda )\;({ℒ}_{algn}+{ℒ}_{bg})$ (Total objective)
**Backward pass:**
Compute gradients $\nabla_{\theta ,\phi }{ℒ}_{SDDA}$
Update parameters: $\theta \leftarrow \theta -\eta \nabla_{\theta }{ℒ}_{SDDA}$
Update parameters: $\phi \leftarrow \phi -\eta \nabla_{\phi }{ℒ}_{SDDA}$
3. **Optional validation:** evaluate on validation set
4. Adjust learning rate or save best model if needed
**Return** $E_{\theta }$, $P_{\phi }$

### 4.1 Datasets

To rigorously assess the efficacy and generalization of SDDA, we performed experiments on five widely used public datasets: FaceForensics++ [5], Celeb-DF [2], Flickr-Faces-HQ (FFHQ) [15], WildDeepfake [16], and ForgeryNet [17]. Collectively, these datasets cover a wide range of Deepfake generation techniques, compression levels, and acquisition conditions, providing a diverse benchmark for evaluating both spatial and frequency-domain anomaly detection capabilities. Brief information about the datasets used in this study is shown in Table 3.

**Table 3:** Statistics of the Deepfake datasets used in this study.

Datasets		Specifications		
Type	Dataset	Format	Resolution	Count
Video-based	FaceForensics++	MP4	720p	1,977 videos / 1,000 sequences
Video-based	Celeb-DF	MP4	256p–720p	5,639 videos
Image-based	FFHQ	PNG	1024×1024	70,000 images
Video-based	WildDeepfake	MP4	480p–1080p	7,314 sequences
Video-based	ForgeryNet	MP4 / JPG	240p–1080p	221,247 videos + 2.9M images

**FaceForensics++ (FF++):** FF++ contains 1,000 original video sequences sourced from YouTube and 977 processed video clips. Each clip typically features a single face with minimal occlusion or motion blur, facilitating stable face tracking and manipulation. Four manipulation methods (*DeepFakes, Face2Face, FaceSwap, NeuralTextures*) are applied to generate corresponding forged videos. For our experiments, we extracted one frame every 10 frames to construct the image dataset.

**Celeb-DF:** Celeb-DF is a challenging large-scale dataset containing diverse facial identities and high-quality manipulations. Compared with previous datasets, Celeb-DF exhibits fewer visual artifacts and more realistic facial dynamics, making it ideal for evaluating model generalization on subtle and unseen forgeries.

**Flickr-Faces-HQ (FFHQ):** FFHQ consists of 70,000 high-resolution (1024×1024) PNG images of real human faces collected from Flickr. The dataset contains substantial diversity in age, ethnicity, facial expression, and illumination conditions, and is therefore well suited for modeling normal facial appearance.

In our anomaly detection setting, FFHQ is used exclusively as a source of *real* samples for training. Since FFHQ does not provide native forged images, fake samples used in the validation and test phases are constructed by incorporating manipulated face images from external Deepfake datasets (e.g., FaceForensics++ and Celeb-DF). These fake samples are *never* used during training, and are only introduced at evaluation time to assess the model’s ability to distinguish real faces from unseen forgeries.

**WildDeepfake:** WildDeepfake comprises 7,314 face sequences from 707 Deepfake videos collected from the internet. It features uncontrolled lighting, compression artifacts, and motion blur, representing real-world challenges for forgery detection. This dataset is primarily used to evaluate SDDA’s robustness in-the-wild.

**ForgeryNet:** ForgeryNet is a large-scale face forgery benchmark containing 1.44 million real images, 1.46 million fake images, and over 220,000 videos from more than 5,400 identities. It includes 15 manipulation methods across face swap, face transfer, face reenactment, and face editing, with 36 perturbation types to simulate real-world distortions. The dataset provides annotations for image and video forgery classification, as well as spatial and temporal forgery localization. In our experiments, we use the image subset and extract one frame every 10 frames to construct the dataset.

**Data Preprocessing and Partitioning:** For video datasets (FF++, Celeb-DF, WildDeepfake, ForgeryNet), frames were uniformly sampled at a rate of 1/10. For image-based datasets (FFHQ), all images were directly used. Data were split into training, validation, and test sets at a 6:2:2 ratio. The training set contains only authentic samples, while validation and test sets include a balanced mix of real and fake samples.

### 4.2 Experimental design and evaluation metrics

#### 4.2.1 Experimental setup.

**Data Preprocessing.** All input face images are first detected and aligned using a standard facial alignment pipeline. The aligned RGB images are resized to a fixed spatial resolution and normalized to [0,1] before being fed into the model. For frequency-domain modeling, we apply a two-dimensional discrete Fourier transform (DFT) to each color channel independently and retain the amplitude spectrum as the spectral representation. To reduce numerical instability, the amplitude spectra are logarithmically scaled and normalized across samples.

**Self-supervised Feature Targets.** High-level semantic feature maps are extracted from a frozen pretrained facial alignment network (FAN). Based on these feature maps, four self-supervised regression targets are constructed, corresponding to local structural deviation, global pattern discrepancy, local residual difference, and total consistency deviation. These targets serve as supervision signals during training and are not used at inference time.

**Network Architecture and Training Details.** The dual-domain encoder adopts a lightweight four-layer convolutional architecture with shared weights across spatial and frequency branches. The specific configuration parameters of the encoder backbone and prediction head are shown in Table 4. Each convolutional layer is followed by LeakyReLU activation and batch normalization. The latent embedding dimension is fixed to *Z* = 8 for all experiments. A shallow multi-layer perceptron (MLP) prediction head is appended to regress the four self-supervised feature targets. All models are trained using the Adam optimizer with a learning rate of $1\times 10^{-3}$ and a batch size of 128. Training is conducted for up to 1000 epochs with early stopping based on validation performance and a patience of 200 epochs. To ensure statistical robustness, all experiments are repeated using six different random seeds, and the mean results are reported.

**Table 4:** Configuration of encoder backbone and prediction head.

Module	Configuration Details
**Encoder Backbone**	Conv2D(in_ch, 32, kernel = 4×4, stride = 2, padding = 1) + LeakyReLU
	Conv2D(32, 64, kernel = 4×4, stride = 2, padding = 1) + BatchNorm + LeakyReLU
	Conv2D(64, 128, kernel = 4×4, stride = 2, padding = 1) + BatchNorm + LeakyReLU
	Conv2D(128, 64, kernel = 2×2, stride = 1, padding = 0) + AdaptiveAvgPool2D + Flatten
**Prediction Head**	Linear(64, 32) + LeakyReLU + Linear(32, 4)

The encoder employs a lightweight convolutional architecture optimized for compact representation learning.

It consists of four sequential 2D convolutional layers with progressively increasing channels, interleaved with non-linear activation and normalization layers. Both spatial-domain RGB images and frequency-domain amplitude spectra are processed in their native two-dimensional form, without reshaping into one-dimensional sequences.

Spatial and frequency branches share identical encoder weights to enforce domain-consistent embeddings. A shallow MLP-based prediction head is appended for multi-feature regression, composed of fully connected layers with LeakyReLU activation.

#### 4.2.2 Evaluation metrics.

The anomaly detection performance was quantified using two widely adopted metrics: **AUC** (Area Under the ROC Curve) and **AP** (Average Precision), in accordance with prior works [9,10]. Each experiment was repeated six times with different random seeds, and both mean and standard deviation values are reported to ensure robustness. A higher AUC indicates better separability between normal and anomalous samples, while a higher AP reflects improved ranking consistency of anomaly scores. Together, these metrics provide a comprehensive evaluation of both model discriminability and generalization across datasets and manipulation types.

### 4.3 Baselines

To comprehensively evaluate the performance of the proposed SDDA framework, we selected twelve state-of-the-art baseline models spanning reconstruction-, adversarial-, flow-, and diffusion-based anomaly detection approaches, which are widely adopted in visual anomaly detection and Deepfake forensics. The details are summarized below:

- **AE-CNN [**8**]** is a classical unsupervised reconstruction-based method that learns to reconstruct normal facial images. Anomalies are detected by measuring pixel-wise reconstruction errors.
- **DeepSVDD [**9**]** is an unsupervised one-class classification approach that maps normal samples to a compact hypersphere in latent space. Samples with large deviations from the hypersphere center are considered anomalous.
- **GANomaly [**10**]** employs an encoder–decoder–encoder architecture to enforce bidirectional feature consistency, using reconstruction and latent-space discrepancies to detect anomalies.
- **AnoGAN [**23**]** reconstructs input images using a pretrained GAN generator via iterative latent-space optimization. The anomaly score combines reconstruction and discriminator losses.
- **DRAEM [**24**]** introduces self-supervised reconstruction using synthetically corrupted images and trains a dual-branch network to restore and discriminate local anomalies.
- **PatchCore [**25**]** leverages memory banks of patch-level embeddings extracted from normal samples. During inference, nearest-neighbor distances are used to detect localized anomalies.
- **FastFlow [**37**]** applies normalizing flows to map high-dimensional visual features into a Gaussian latent space, enabling efficient detection and localization based on feature density.
- **CutPaste [**38**]** is a self-supervised augmentation technique that generates synthetic anomalies via patch cutting and pasting, training models to distinguish real from altered images.
- **Reverse Distillation [**36**]** employs a teacher–student self-distillation mechanism where reconstruction errors in intermediate feature layers indicate anomaly likelihood.
- **CFlow [**39**]** utilizes conditional normalizing flows to estimate feature-space likelihoods, achieving high efficiency and real-time performance for facial anomaly detection.
- **FATNet [**34**]** is a frequency-aware transformer tailored for Deepfake detection, leveraging dual-domain joint attention to capture subtle cross-domain manipulation traces.
- **DiffusionAD [**35**]** is a diffusion-based anomaly detection model that iteratively denoises input images to reconstruct clean samples, enabling robust detection of subtle manipulations.
- **RepDFD [**49**]** repurposes a pre-trained Vision-Language Model (e.g., CLIP) for deepfake detection without altering the model’s internal parameters. It utilizes learnable visual perturbations and adaptive text prompts to guide the optimization process, significantly improving generalization and efficiency in deepfake detection.

All baseline models were trained under identical data splits and preprocessing pipelines to ensure fair comparison. Only authentic (non-manipulated) facial data were used during training, while both real and fake samples were employed for validation and testing.

### 4.4 Experimental results

#### 4.4.1 Performance analysis.

In this section, we quantitatively evaluate the anomaly detection performance of our proposed SDDA framework. The results of all the compared methods on the five benchmark datasets are summarized in Tables 5 and 6. The proposed SDDA consistently achieves the highest AUC and AP values across all datasets, demonstrating superior generalization to diverse forgery types and compression levels. On the **FaceForensics** dataset, SDDA attains an AUC of **82.36%** and an AP of **80.41%**, outperforming the strongest baseline DiffusionAD by 7.71% and 13.01%, respectively. On the more challenging **Celeb-DF** dataset, which contains complex post-processing and realistic facial reenactments, SDDA achieves **81.56%** AUC and **78.47%** AP, surpassing DiffusionAD by 13.84% in AUC and 11.58% in AP, indicating its robustness under strong compression and illumination variations. In the **Flickr-Faces-HQ** dataset, SDDA reaches **73.78%** AUC and **71.38%** AP, improving over the best-performing baseline by 18.44% in AUC and 9.26% in AP, highlighting its capability to capture fine-grained local frequency inconsistencies. Similarly, on the **WildDeepfake** dataset, which includes uncontrolled in-the-wild conditions, SDDA achieves **76.45%** AUC and **72.31%** AP, exceeding DiffusionAD by 9.33% and 8.09%, respectively.

**Table 5:** Anomaly detection performance (AUC) of all methods.The best results are marked in bold, and all values are percentages (%). Results are reported as mean ± standard deviation across six random seeds.

Method	FaceForensics	Celeb-DF	Flickr-Faces-HQ	WildDeepfake	ForgeryNet
AE-CNN	53.68 ± 5.10	65.52 ± 0.15	50.62 ± 0.30	45.74 ± 0.62	67.84 ± 2.21
DeepSVDD	58.05 ± 3.42	60.94 ± 0.10	48.00 ± 0.07	59.37 ± 0.71	74.21 ± 0.20
GANomaly	63.98 ± 0.29	65.20 ± 1.20	50.67 ± 0.40	46.93 ± 0.10	69.11 ± 1.42
PaDiM	67.73 ± 0.92	56.13 ± 1.11	50.53 ± 0.22	45.74 ± 0.63	71.43 ± 1.71
FastFlow	58.52 ± 0.97	64.35 ± 0.30	50.28 ± 0.18	50.18 ± 0.43	75.04 ± 0.18
DRAEM	56.62 ± 0.03	51.36 ± 0.03	51.01 ± 0.01	55.11 ± 0.30	68.37 ± 2.15
CFlow	69.94 ± 1.08	64.53 ± 0.46	50.20 ± 0.03	50.43 ± 1.15	80.86 ± 0.11
CutPaste	57.93 ± 0.59	52.36 ± 0.65	50.60 ± 0.50	59.33 ± 0.16	73.56 ± 0.16
PatchCore	63.07 ± 0.27	61.74 ± 0.32	50.47 ± 0.08	53.75 ± 0.41	70.26 ± 2.19
RD4AD	67.37 ± 0.80	65.76 ± 0.36	50.34 ± 0.05	58.57 ± 0.95	62.08 ± 1.22
FATNet	72.83 ± 3.46	62.34 ± 2.11	55.56 ± 0.36	66.28 ± 0.87	81.44 ± 1.53
DiffusionAD	74.65 ± 0.97	67.72 ± 0.55	55.34 ± 0.02	67.12 ± 0.25	65.92 ± 2.70
RepDFD	65.25 ± 0.10	74.20 ± 0.37	62.40 ± 0.11	71.30 ± 0.60	70.42 ± 2.33
SDDA	**82.36 ± 2.69**	**81.56 ± 0.49**	**73.78 ± 0.65**	**76.45 ± 1.15**	**83.13 ± 1.12**

**Table 6:** Anomaly detection performance (AP) of all methods.The best results are marked in bold, and all values are percentages (%). Results are reported as mean ± standard deviation across six random seeds.

Method	FaceForensics	Celeb-DF	Flickr-Faces-HQ	WildDeepfake	ForgeryNet
AE-CNN	54.35 ± 5.60	64.78 ± 0.09	50.26 ± 0.13	47.07 ± 0.48	63.26 ± 0.16
DeepSVDD	56.05 ± 2.81	60.74 ± 0.32	48.28 ± 0.24	57.15 ± 0.47	70.54 ± 2.50
GANomaly	54.60 ± 0.39	64.59 ± 0.74	50.14 ± 0.10	47.71 ± 0.05	62.45 ± 4.11
PaDiM	60.76 ± 0.92	58.10 ± 0.97	49.72 ± 0.24	47.06 ± 0.50	70.23 ± 2.40
FastFlow	59.33 ± 1.25	64.16 ± 0.45	50.14 ± 0.36	51.63 ± 0.70	71.20 ± 0.42
DRAEM	57.18 ± 0.02	52.47 ± 0.03	50.81 ± 0.02	55.05 ± 0.13	66.23 ± 0.46
CFlow	60.83 ± 0.98	65.07 ± 0.36	50.05 ± 0.20	51.91 ± 0.64	78.33 ± 0.23
CutPaste	58.01 ± 0.41	53.52 ± 0.38	50.32 ± 0.14	57.35 ± 0.10	74.57 ± 0.52
PatchCore	63.52 ± 0.43	60.29 ± 0.43	50.18 ± 0.16	56.27 ± 0.37	70.10 ± 0.30
RD4AD	58.07 ± 0.67	65.88 ± 0.59	50.27 ± 0.10	59.77 ± 1.04	59.41 ± 0.32
FATNet	65.76 ± 2.89	61.18 ± 0.98	63.77 ± 0.21	62.19 ± 1.02	77.60 ± 0.47
DiffusionAD	67.40 ± 0.68	66.89 ± 1.26	62.12 ± 0.54	64.22 ± 0.55	60.94 ± 1.35
RepDFD	61.45 ± 0.26	72.04 ± 0.20	66.42 ± 0.28	65.05 ± 0.52	72.51 ± 0.89
SDDA	**80.41 ± 1.55**	**78.47 ± 2.58**	**71.38 ± 0.47**	**72.31 ± 0.95**	**82.40 ± 1.37**

These results clearly demonstrate the effectiveness of the proposed dual-domain self-supervised learning paradigm. By jointly modeling spatial and frequency representations through shared-weight encoders, SDDA captures both global structural patterns and local spectral inconsistencies, enabling stronger discrimination of subtle manipulations. Unlike conventional autoencoder- or GAN-based frameworks that focus solely on spatial reconstruction, SDDA introduces a self-supervised feature prediction strategy that estimates four domain-consistent descriptors (ℓ, γ, ρ, τ), thereby enhancing the semantic alignment between the spatial and frequency domains. This design not only improves detection accuracy but also enhances interpretability by linking feature deviations to specific spectral or spatial distortions.

Furthermore, SDDA achieves superior performance without relying on labeled fake data or handcrafted frequency fusion. This property underscores its potential for real-world deployment in open-world deepfake forensics, where unseen manipulation types frequently emerge. Overall, SDDA achieves a robust balance between discriminability, generalization, and interpretability, outperforming existing state-of-the-art deepfake anomaly detection methods across all evaluation benchmarks.

#### 4.4.2 Ablation study.

To evaluate the contribution of each key component in the proposed SDDA framework, we conduct comprehensive ablation experiments on all five benchmark datasets: FaceForensics++, Celeb-DF, Flickr-Faces-HQ, WildDeepfake, and ForgeryNet. Five ablation variants are designed by selectively removing individual modules or objectives, as described below:

- **wo-***F*: The frequency-domain branch is removed, retaining only the spatial encoder, thereby eliminating the spatial–spectral consistency constraint.
- **wo-***SD*: The *Local Structural Deviation (LSD)* prediction task is disabled, preventing the model from enforcing local structural alignment between spatial and frequency representations.
- **wo-***PD*: The *Global Pattern Discrepancy (GPD)* objective is removed, weakening the learning of global structural consistency.
- **wo-***RD*: The *Local Residual Difference (LRD)* predictor is excluded, reducing the model’s sensitivity to fine-grained local anomalies.
- **wo-***TD*: The *Total Consistency Deviation (TCD)* constraint is removed, disabling unified cross-domain consistency calibration.

As presented in Tables 7 and 8, the removal of any individual module results in a noticeable degradation in both AUC and AP across all datasets, confirming the contribution of each component to the overall performance of SDDA. The most substantial decline occurs when the **frequency-domain branch** is removed, with average decreases of 12.83% (AUC) and 12.75% (AP) across datasets, highlighting the critical role of frequency-domain cues in capturing subtle spectral artifacts introduced by manipulations. Among the four self-supervised predictive features, eliminating *TCD* leads to the second-largest performance drop (average decreases of 8.84% AUC and 8.65% AP), underscoring its importance in integrating cross-domain information. *LSD* and *GPD* primarily contribute to learning local and global structural patterns, whereas *LRD* and *TCD* reinforce consistency-based discrimination. Overall, the complete SDDA framework achieves the highest AUC and AP across all datasets, demonstrating that the collaborative effect of all components enhances dual-domain feature alignment, anomaly sensitivity, and the compactness and discriminability of learned representations.

**Table 7:** Ablation study of the contribution of each module in SDDA (AUC). The best results are marked in bold, and all values are percentages (%). Results are reported as mean ± standard deviation across six random seeds.

Variant	FaceForensics	Celeb-DF	Flickr-Faces-HQ	WildDeepfake	ForgeryNet
wo-*F*	73.86 ± 1.45	69.84 ± 1.14	59.02 ± 0.65	60.12 ± 0.81	70.40 ± 0.23
wo-*L*	75.34 ± 2.03	70.35 ± 0.95	60.55 ± 0.49	61.24 ± 0.97	66.54 ± 0.54
wo-*G*	76.12 ± 1.78	70.78 ± 0.77	61.27 ± 0.58	62.05 ± 0.72	73.72 ± 0.40
wo-*R*	77.24 ± 1.56	71.34 ± 0.89	62.03 ± 0.64	63.22 ± 0.78	75.26 ± 1.15
wo-*T*	78.32 ± 1.34	72.45 ± 0.92	62.84 ± 0.56	65.17 ± 0.75	70.23 ± 0.69
**SDDA**	**82.36 ± 2.69**	**81.56 ± 0.49**	**73.78 ± 0.65**	**76.45 ± 1.15**	**83.13 ± 1.12**

**Table 8:** Ablation study of the contribution of each module in SDDA (AP).The best results are marked in bold, and all values are percentages (%). Results are reported as mean ± standard deviation across six random seeds.

Variant	FaceForensics	Celeb-DF	Flickr-Faces-HQ	WildDeepfake	ForgeryNet
wo-*F*	70.22 ± 1.31	66.05 ± 0.93	57.28 ± 0.52	58.03 ± 0.63	71.13 ± 1.05
wo-*SD*	72.09 ± 1.47	67.12 ± 0.88	58.61 ± 0.74	59.10 ± 0.69	74.20 ± 0.41
wo-*PD*	73.01 ± 1.30	68.22 ± 0.65	59.33 ± 0.63	60.21 ± 0.55	70.50 ± 1.27
wo-*RD*	74.12 ± 1.25	68.85 ± 0.82	60.41 ± 0.59	60.84 ± 0.62	76.73 ± 0.42
wo-*TD*	75.65 ± 1.17	69.54 ± 0.77	60.96 ± 0.68	61.83 ± 0.58	73.51 ± 1.22
**SDDA**	**80.41 ± 1.55**	**78.47 ± 2.58**	**71.38 ± 0.47**	**72.31 ± 0.95**	**82.40 ± 1.37**

#### 4.4.3 Generalization experiments.

In this section, we evaluate the generalization performance of the SDDA model. We used data from the ForgeryNet dataset as the training dataset and the FaceForensics and Celeb-DF datasets as the test datasets. Similarly, we used data from the Flickr-Faces-HQ dataset as the training dataset and the WildDeepfake dataset as the test dataset. The results of the generalization experiments are shown in the Table 9 below.

**Table 9:** Anomaly detection performance in generalization experiments. All values are percentages(%) and the best results are marked in bold.

Methods	ForgeryNet → FaceForensics		ForgeryNet → Celeb-DF		Flickr-Faces-HQ → WildDeepfake	
	AP	AUC	AP	AUC	AP	AUC
DeepSVDD	60.20 ± 3.22	59.45 ± 2.24	64.27 ± 0.23	61.44 ± 0.50	67.20 ± 0.48	65.15 ± 0.29
CFlow	67.05 ± 1.23	58.91 ± 0.17	62.99 ± 0.26	63.24 ± 0.16	50.33 ± 0.98	51.47 ± 0.20
CutPaste	60.37 ± 0.53	59.33 ± 0.26	54.20 ± 1.73	51.42 ± 1.45	54.21 ± 0.61	53.62 ± 1.32
FATNet	73.38 ± 2.68	66.50 ± 1.78	63.71 ± 1.27	59.97 ± 1.17	64.25 ± 0.15	65.31 ± 0.58
RepDFD	69.70 ± 0.25	65.47 ± 0.75	72.35 ± 2.56	71.08 ± 0.43	68.18 ± 0.41	67.62 ± 1.57
**SDDA**	**75.15 ± 2.20**	**76.56 ± 0.62**	**75.47 ± 0.32**	**74.07 ± 1.46**	**70.26 ± 0.97**	**68.17 ± 0.83**

The results show that the SDDA model exhibits superior generalization performance compared to the models listed. Overall, when trained on the ForgeryNet dataset and tested on the Celeb-DF dataset, the SDDA model maintains good performance, outperforming other comparative models. However, when trained on the Flickr-Faces-HQ dataset and tested on the WildDeepfake dataset, the performance of the SDDA model decreases compared to training and testing directly on the WildDeepfake dataset. Nevertheless, this model still outperforms other comparative models. We speculate that the performance degradation is due to significant data differences between the Flickr-Faces-HQ dataset, which primarily uses images, and the WildDeepfake dataset, which processes videos into images.

For visualization, we selected four representative baseline methods: DeepSVDD, PatchCore, DiffusionAD, and SDDA. These methods represent three major paradigms in anomaly detection: hypersphere-based (DeepSVDD), memory-anchored patch reasoning (PatchCore), diffusion-driven reconstruction (DiffusionAD), and our proposed dual-domain self-supervised feature alignment framework (SDDA). To ensure a comprehensive evaluation, we conducted visual analyses across five benchmark datasets: FaceForensics++, Celeb-DF, Flickr-Faces-HQ (FFHQ), WildDeepfake, and ForgeryNet. This setup offers an extensive view of how each model generalizes to various real-world manipulation scenarios. While the quantitative superiority of SDDA has been confirmed in previous experiments, the visualization results further demonstrate that SDDA produces more compact, structured, and separable representations compared to the other representative baselines.

**Fig 3:**
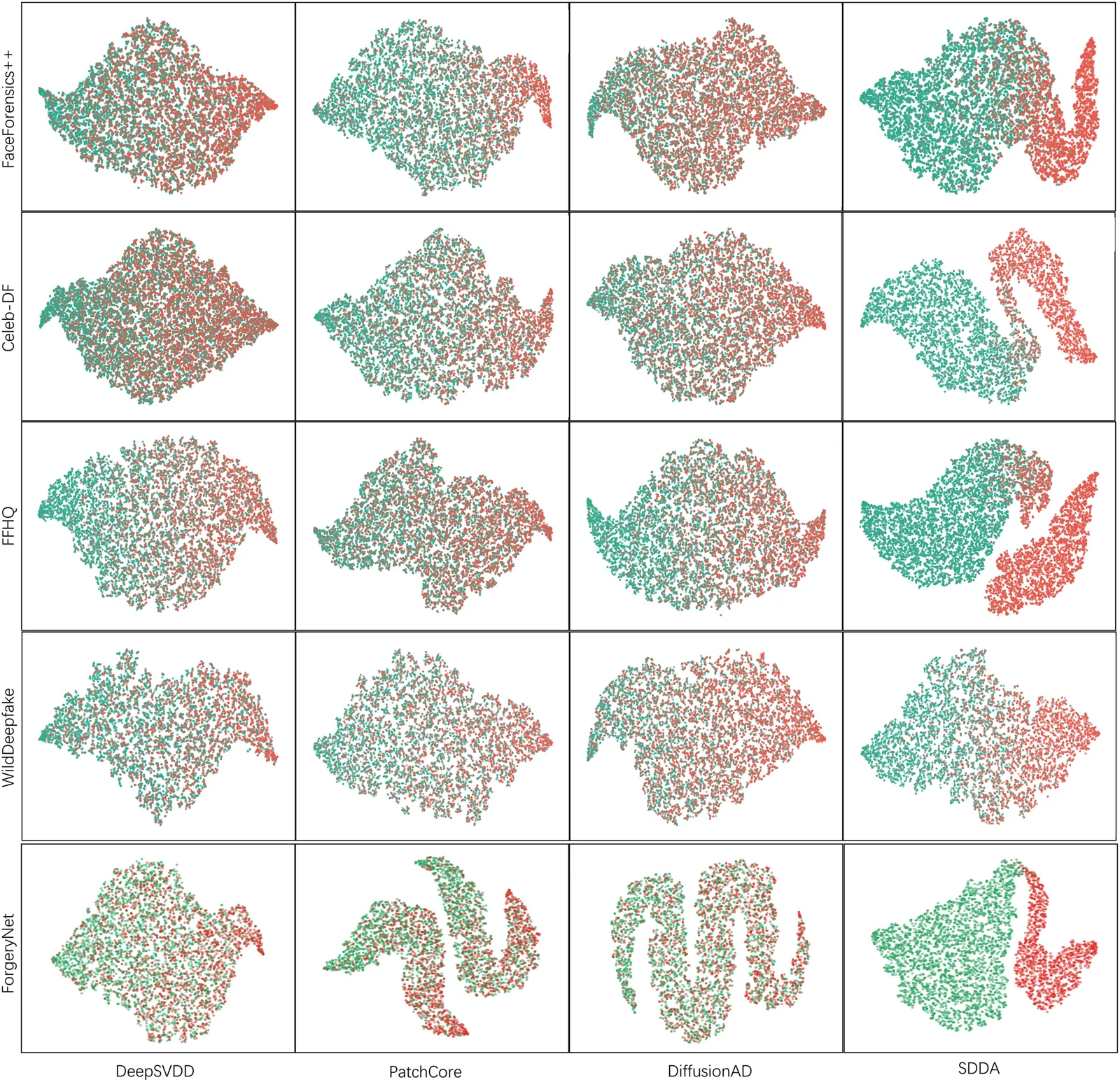
Feature space embedding visualization of SDDA and compared methods (DeepSVDD, PatchCore, and DiffusionAD) across five datasets: FaceForensics++, Celeb-DF, FFHQ, WildDeepfake, and ForgeryNet. Green points represent real (normal) samples, and red points represent fake (abnormal) samples. SDDA produces the most compact and separable clusters across all datasets, highlighting its superior dual-domain representation alignment and anomaly discriminability.

**Fig 4:**
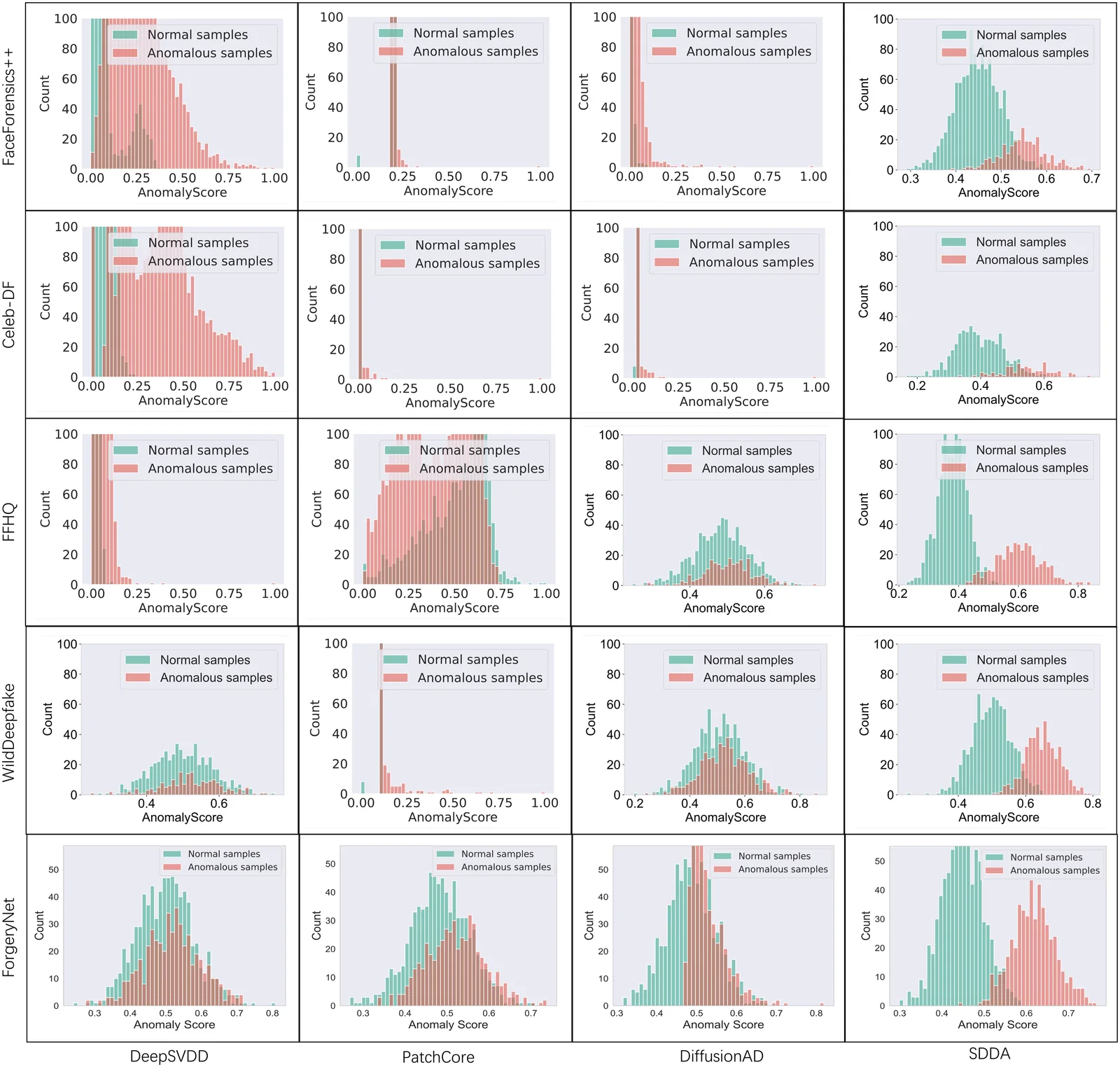
Anomaly scores visualization of SDDA and compared methods (DeepSVDD, PatchCore, and DiffusionAD) across five datasets: FaceForensics++, Celeb-DF, FFHQ, WildDeepfake, and ForgeryNet. Green bars represent real (normal) samples, and red bars represent fake (abnormal) samples. SDDA produces the most compact and separable clusters across all datasets, highlighting its superior dual-domain representation alignment and anomaly discriminability.

- **Feature embedding visualization and analysis:** To assess the discriminability of the learned representations, we visualized the latent embeddings of the test samples using the t-SNE algorithm. As illustrated in Fig 3, green points correspond to real (authentic) samples, while red points denote fake (manipulated) samples. The visualization is conducted separately on five datasets—FaceForensics++, Celeb-DF, FFHQ, WildDeepfake, and ForgeryNet—and the results are compared across four methods: DeepSVDD, PatchCore, DiffusionAD, and SDDA (from left to right). The embeddings of DeepSVDD show weak separation between normal and fake regions, indicating that hypersphere mapping struggles to capture complex facial manipulations. PatchCore demonstrates moderate clustering improvement due to local patch memory matching but still suffers from overlapping regions between normal and abnormal points. DiffusionAD generates smoother embeddings yet exhibits partial entanglement between fake and real clusters, reflecting its diffusion-driven stochastic reconstruction behavior. In contrast, SDDA forms distinct, compact clusters with clearly delineated boundaries between authentic and manipulated samples. This result confirms that the proposed dual-domain feature alignment and self-supervised prediction module effectively enhance both representation compactness and discrimination capability across diverse datasets.
- **Anomaly distribution visualization and analysis:** To further analyze the separability of normal and abnormal samples, we visualize the anomaly score distributions obtained by the four methods on the same datasets, as shown in Fig 4. Each model computes anomaly scores using its respective mechanism—distance from the hypersphere center (DeepSVDD), feature deviation from memory patches (PatchCore), reconstruction residuals (DiffusionAD), and the unified global–local deviation score (SDDA). In these histograms, green bars indicate normal samples and red bars indicate fake samples. The results show that SDDA exhibits two clearly separated peaks with minimal overlap, whereas DeepSVDD and PatchCore yield wide overlapping areas, and DiffusionAD presents a partially mixed distribution due to its generative noise. SDDA’s sharp boundary between normal and fake score distributions demonstrates its superior capability to learn consistent, domain-aligned representations and accurate anomaly boundaries, making it robust against unseen manipulations across all five benchmark datasets.

#### 4.4.4 Parameter sensitivity analysis.

We conducted a parameter sensitivity analysis to evaluate the influence of the weighting coefficient λ in the SDDA loss function (Eq. 16) on anomaly detection performance across the five benchmark datasets. The coefficient λ balances the contribution between the feature prediction loss and the combined domain alignment and hypersphere compactness losses.

**Fig 5:**
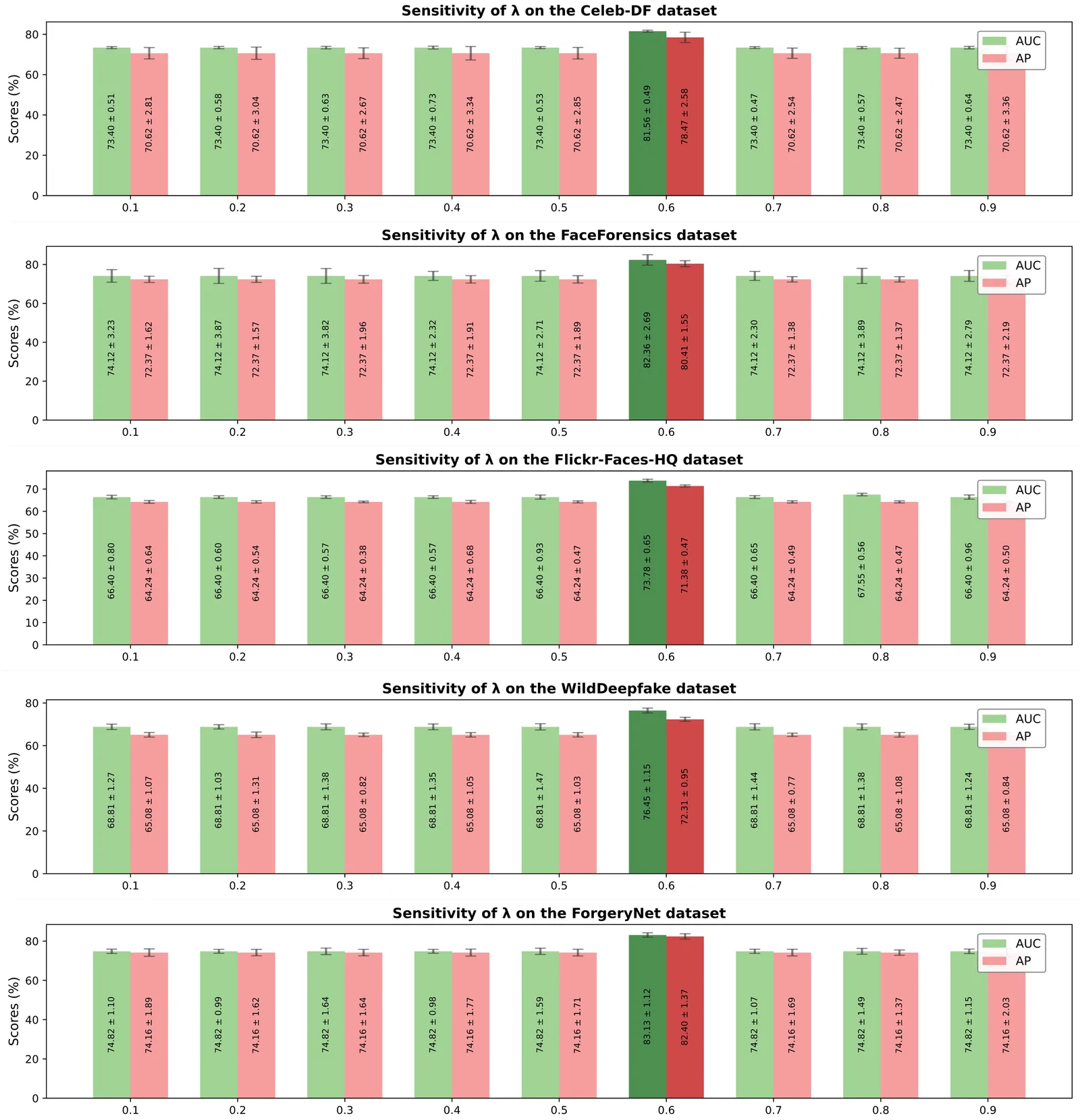
Parameter sensitivity analysis of SDDA on the five datasets: FaceForensics, Celeb-DF, Flickr-Faces-HQ, WildDeepfake, and ForgeryNet.

As shown in Fig 5, SDDA achieves optimal performance on the FaceForensics++, Celeb-DF, and FFHQ datasets when λ=0.5, indicating a balanced trade-off between feature prediction and domain alignment objectives yields the best discriminability and generalization. For the WildDeepfake dataset, which contains highly diverse in-the-wild conditions, the best performance is observed at λ=0.6, suggesting a slightly higher emphasis on the feature prediction loss is beneficial under more challenging scenarios. Overall, the results demonstrate that SDDA exhibits stable performance across a reasonable range of λ values, confirming the robustness of the proposed dual-domain self-supervised learning framework to hyperparameter variations.

### 4.5 Computational analysis

We further performed a computational complexity analysis to assess the efficiency and deployability of SDDA in practical deepfake anomaly detection scenarios. Table 10 summarizes the number of parameters, FLOPs, and average training/testing time per epoch and per batch for three representative baseline models and our SDDA framework. The results indicate that SDDA maintains a moderate parameter size (0.96 MB) and computational cost (4.12 G FLOPs), which is significantly lower than that of DiffusionAD (7.85 G) while providing faster inference (0.78 s). Although PatchCore achieves slightly faster testing speed (0.65 s), it suffers from lower feature expressiveness due to its non-learnable memory bank design. Overall, SDDA achieves a balanced trade-off between accuracy and computational efficiency, making it suitable for real-time deployment in resource-constrained or embedded deepfake forensics applications.

**Table 10:** Comparison of computational complexity and efficiency across different anomaly detection methods. SDDA achieves competitive performance with moderate computational overhead.

Method	Params (MB)	FLOPs (G)	Train (s)	Test (s)
DeepSVDD	0.52	1.84	21.63	1.12
PatchCore	0.37	3.25	18.79	0.65
DiffusionAD	1.82	7.85	35.94	1.46
SDDA (Ours)	0.96	4.12	24.57	0.78

### 4.6 Failure cases and limitations

Despite the strong empirical performance of SDDA across multiple benchmarks, several limitations merit further discussion. One limitation arises when the input faces suffer from severe quality degradation, such as heavy compression, strong motion blur, or extreme downsampling. Under these conditions, both spatial textures and frequency amplitudes may be significantly distorted, thereby weakening the effectiveness of spatial–frequency consistency modeling. In addition, identity-preserving manipulations that introduce only subtle or highly localized artifacts remain challenging. Advanced facial reenactment techniques that closely mimic authentic facial statistics may exhibit limited spectral deviation, reducing the anomaly margin learned during self-supervised training. Moreover, the adopted FFT-based frequency representation primarily captures global spectral characteristics. While this design is effective for identifying common generative artifacts, it may be less sensitive to localized or non-periodic distortions. Incorporating multi-scale or adaptive frequency decomposition strategies could further enhance sensitivity to fine-grained anomalies. Finally, the hypersphere-based anomaly modeling assumes a relatively compact distribution of normal samples in the latent space. Although this assumption works well in practice, it may restrict expressiveness when normal data exhibit complex or multi-modal distributions. Exploring more flexible density modeling approaches constitutes an important direction for future research.

## Conclusion

In this work, we introduced **SDDA**, a novel dual-domain feature alignment framework for deepfake anomaly detection. Unlike conventional supervised or reconstruction-based approaches, SDDA leverages a self-supervised learning paradigm that jointly enforces spatial-frequency consistency while predicting multiple discriminative features, without requiring any forged samples during training. Extensive experiments on five benchmark datasets demonstrate that SDDA consistently outperforms state-of-the-art anomaly detection methods in both AUC and AP metrics. These results highlight its superior robustness, generalization to unseen manipulations, and sensitivity to subtle spatial and spectral discrepancies. Our findings validate the effectiveness of explicitly aligning spatial and spectral representations for anomaly detection and underscore the potential of dual-domain self-supervised learning as a foundation for future research. This framework opens avenues for developing more efficient, interpretable, and scalable deepfake detection systems, particularly in real-world and open-world scenarios.
