# Supplementary material for: Dual-domain self-supervised feature alignment via spectral–spatial representation learning for deepfake anomaly detection
Source: PLoS One. 2026 Sep 11;21(9):e0358049. doi: 10.1371/journal.pone.0358049 (PMC13568503; doi:10.1371/journal.pone.0358049)
Supplement: Supplementary file 1 [file pone.0358049.s002.pdf]

Response to Reviewers

Manuscript ID: PONE-D-25-63428

Title: *Dual-Domain Self-Supervised Feature Alignment via Spectral–Spatial Representation Learning for Deepfake Anomaly Detection*

Response to the Academic Editor

We sincerely thank the Academic Editor for the careful handling of our manuscript and for the constructive suggestions.

In response to the editor's comments, we have thoroughly revised the manuscript to ensure full compliance with PLOS ONE formatting and submission requirements. Specifically, we improved the clarity of the method description, standardized terminology across sections, corrected figure and table references, and refined the dataset descriptions. All modifications to the manuscript are explicitly marked in blue in the revised version.

Below are our point-by-point responses to the journal requirements and a summary of the revisions made to the manuscript:

Journal Requirements:

1. **Style Requirements:** We have carefully reformatted the manuscript using the provided PLOS ONE style templates to ensure full compliance with all formatting guidelines, including file naming conventions.

2. **Code Sharing:** As our work relies on author-generated code, we commit to sharing it upon publication. We have prepared our code for deposition in a public repository (e.g., GitHub or Zenodo) and will provide the repository URL and DOI in the final manuscript's Data Availability Statement.

3. **Data Availability Statement:** All data used in this study are from publicly available datasets, and the corresponding references have been provided in the article. The URL corresponding to the dataset is as follows:

   FaceForensics++: https://github.com/ondyari/FaceForensics.

   Celeb-DF: https://github.com/yuezunli/celeb-deepfakeforensics.

   Flickr-Faces-HQ: https://github.com/NVlabs/ffhq-dataset.

   WildDeepfake: https://github.com/OpenTAI/wild-deepfake.

4. **ORCID iD:** We confirm that the corresponding author's ORCID iD has been validated in the Editorial Manager system.

5. **Consent for Publication of Identifiable Images:** All images used in the manuscript are AI-generated.

6. **Reference to Tables:** We have carefully reviewed the manuscript and have now inserted the missing citations for **Tables 2 through 8** in the relevant results and discussion sections.

7. **Reviewer Citation Recommendations:** We have reviewed the relevant publications suggested by the reviewers. Appropriate citations have been added to the revised manuscript where they provide relevant context or support for our methodology and discussion, as indicated in our detailed 'Response to Reviewers' file.

Revisions to the Manuscript:

Beyond addressing the specific requirements listed above, we have thoroughly revised the manuscript in response to the reviewers' feedback. Key improvements include enhanced clarity in the methodology section, standardized terminology, and refined dataset descriptions. All textual changes are highlighted in blue in the 'Revised Manuscript with Track Changes' file.

We believe these revisions have significantly strengthened the manuscript and fully address PLOS ONE's criteria. Thank you again for your guidance.

Response to Reviewer #1

We sincerely thank Reviewer #1 for the careful reading of our manuscript and for the constructive comments that helped improve the clarity, consistency, and technical rigor of the paper. Below, we respond to each comment point by point.

Comment 1

Several baseline names are written inconsistently (e.g., CFlow vs. CFLow, AECNN vs. AE-CNN). Please standardize the naming throughout the manuscript for clarity.

Response

We thank the reviewer for pointing out this issue. We have carefully checked all baseline method names throughout the manuscript and standardized their notation to ensure consistency and clarity.

Revised manuscript text

4.4 Experimental results

...

Comment 2

In the Introduction section, the manuscript lists FF++, CelebDF, DFDC, and DeeperForensics as the experimental datasets, whereas the Experiments section actually uses FF++, CelebDF, FFHQ, and WildDeepfake. Dataset description is inconsistent between sections.

Response

We appreciate the reviewer for identifying this inconsistency. We have revised the Introduction to ensure that the dataset descriptions exactly match those used in the experimental section.

Table 5: **Anomaly detection performance (AUC) of all methods.The best results are marked in bold, and all values are percentages (%). Results are reported as mean ± standard deviation across six random seeds.**

**Method**	**FaceForensics**	**Celeb-DF**	**Flickr-Faces-HQ**	**WildDeepfake**
AE-CNN	53.68 ± 5.10	65.52 ± 0.15	50.62 ± 0.30	45.74 ± 0.62
DeepSVDD	58.05 ± 3.42	60.94 ± 0.10	48.00 ± 0.07	59.37 ± 0.71
GANomaly	63.98 ± 0.29	65.20 ± 1.20	50.67 ± 0.40	46.93 ± 0.10
PaDiM	67.73 ± 0.92	56.13 ± 1.11	50.53 ± 0.22	45.74 ± 0.63
FastFlow	58.52 ± 0.97	64.35 ± 0.30	50.28 ± 0.18	50.18 ± 0.43
DRAEM	56.62 ± 0.03	51.36 ± 0.03	51.01 ± 0.01	55.11 ± 0.30
CFlow	69.94 ± 1.08	64.53 ± 0.46	50.20 ± 0.03	50.43 ± 1.15
CutPaste	57.93 ± 0.59	52.36 ± 0.65	50.60 ± 0.50	59.33 ± 0.16
PatchCore	63.07 ± 0.27	61.74 ± 0.32	50.47 ± 0.08	53.75 ± 0.41
RD4AD	67.37 ± 0.80	65.76 ± 0.36	50.34 ± 0.05	58.57 ± 0.95
FATNet	72.83 ± 3.46	62.34 ± 2.11	55.56 ± 0.36	66.28 ± 0.87
DiffusionAD	74.65 ± 0.97	67.72 ± 0.55	55.34 ± 0.02	67.12 ± 0.25
RepDFD	65.25 ± 0.10	74.20 ± 0.37	62.40 ± 0.11	71.30 ± 0.60
SDDA	**82.36 ± 2.69**	**81.56 ± 0.49**	**73.78 ± 0.65**	**76.45 ± 1.15**

Table 6: **Anomaly detection performance (AP) of all methods.The best results are marked in bold, and all values are percentages (%). Results are reported as mean ± standard deviation across six random seeds.**

**Method**	**FaceForensics**	**Celeb-DF**	**Flickr-Faces-HQ**	**WildDeepfake**
AE-CNN	54.35 ± 5.60	64.78 ± 0.09	50.26 ± 0.13	47.07 ± 0.48
DeepSVDD	56.05 ± 2.81	60.74 ± 0.32	48.28 ± 0.24	57.15 ± 0.47
GANomaly	54.60 ± 0.39	64.59 ± 0.74	50.14 ± 0.10	47.71 ± 0.05
PaDiM	60.76 ± 0.92	58.10 ± 0.97	49.72 ± 0.24	47.06 ± 0.50
FastFlow	59.33 ± 1.25	64.16 ± 0.45	50.14 ± 0.36	51.63 ± 0.70
DRAEM	57.18 ± 0.02	52.47 ± 0.03	50.81 ± 0.02	55.05 ± 0.13
CFlow	60.83 ± 0.98	65.07 ± 0.36	50.05 ± 0.20	51.91 ± 0.64
CutPaste	58.01 ± 0.41	53.52 ± 0.38	50.32 ± 0.14	57.35 ± 0.10
PatchCore	63.52 ± 0.43	60.29 ± 0.43	50.18 ± 0.16	56.27 ± 0.37
RD4AD	58.07 ± 0.67	65.88 ± 0.59	50.27 ± 0.10	59.77 ± 1.04
FATNet	65.76 ± 2.89	61.18 ± 0.98	63.77 ± 0.21	62.19 ± 1.02
DiffusionAD	67.40 ± 0.68	66.89 ± 1.26	62.12 ± 0.54	64.22 ± 0.55
RepDFD	61.45 ± 0.26	72.04 ± 0.20	66.42 ± 0.28	65.05 ± 0.52
SDDA	**80.41 ± 1.55**	**78.47 ± 2.58**	**71.38 ± 0.47**	**72.31 ± 0.95**

Revised manuscript text

Introduction

...

We evaluate our framework on four public datasets—FaceForensics++ [5], Celeb-DF [2], Flickr-Faces-HQ [16], and WildDeepfake [17]. Extensive experiments demonstrate that our method consistently surpasses state-of-the-art baselines, yielding substantial improvements in AUC and average precision (AP).

Comment 3

The manuscript contains multiple placeholders (e.g., "Table ??") and even "placeholder SA" in the references. These must be removed before resubmission.

Response

We thank the reviewer for noting these oversights. All placeholders have been carefully removed, and missing references, table numbers, and citations have been fully corrected.

Revised manuscript text

4.4.1 Performance analysis

...

The results of all the compared methods on the four benchmark datasets are summarized in Table 5 and Table 6.

"4.4.2 Ablation study

...

As presented in Table [7] and Table [8], the removal of any individual module results in a noticeable degradation in both AUC and AP across all datasets, confirming the contribution of each component to the overall performance of SDDA.

Comment 4

The symbols I_s, I_f, I_s, I_f are not clearly defined in the main text. Please explicitly specify whether they refer to the raw images, frequency spectra, or FAN feature maps.

Response

We thank the reviewer for this important clarification request. We have explicitly defined all input symbols at their first occurrence in the Method section.

Revised manuscript text

3.2 Data Preprocessing: Semantic Feature Extraction and Spectral Transformation

Throughout this paper, we denote the input face image in the spatial (RGB) domain as $\mathbf{X}_s \in \mathbb{R}^{H\times W\times 3}$. Its corresponding frequency-domain representation, obtained via a two-dimensional discrete Fourier transform (DFT), is denoted as $\mathbf{X}_f$. Unless otherwise specified, $\mathbf{X}_s$ and $\mathbf{X}_f$ always refer to raw spatial images and their frequency spectra, respectively. Feature maps extracted by the pretrained FAN network are explicitly denoted as $\mathbf{F}_s$ and $\mathbf{F}_f$ to avoid ambiguity.

Comment 5

FFHQ is introduced as a dataset containing only real images, yet the experimental setup states that the validation and test sets are balanced mixtures of real and fake samples. Since FFHQ

does not provide a native fake subset, the source and construction of the fake samples must be clearly described.

Response

We appreciate the reviewer for raising this critical point. We have clarified that FFHQ is used exclusively as a real-only dataset, and fake samples are sourced from external Deepfake datasets during evaluation.

Revised manuscript text

4.1 Datasets

...

Flickr-Faces-HQ (FFHQ): FFHQ consists of 70,000 high-resolution (1024×1024) PNG images of real human faces collected from Flickr. The dataset contains substantial diversity in age, ethnicity, facial expression, and illumination conditions, and is therefore well suited for modeling normal facial appearance. In our anomaly detection setting, FFHQ is used exclusively as a source of *real* samples for training. Since FFHQ does not provide native forged images, fake samples used in the validation and test phases are constructed by incorporating manipulated face images from external Deepfake datasets (e.g., FaceForensics++ and Celeb-DF). These fake samples are *never* used during training, and are only introduced at evaluation time to assess the model's ability to distinguish real faces from unseen forgeries.

Comment 6

The contributions emphasize a "unified anomaly scoring mechanism" combining global reconstruction errors with local frequency-domain prediction discrepancies. However, Section 3.8 defines the anomaly score solely as the sum of distances to the center c (Eq. 11), with no reconstruction branch or score fusion explicitly specified.

Response

We thank the reviewer for identifying this inconsistency. We have revised the anomaly scoring description to clearly reflect the unified scoring mechanism combining global representation compactness and local feature prediction deviation.

Revised manuscript text

3.8 Anomaly Scoring

The final anomaly score integrates both global hypersphere deviation and local self-supervised feature prediction error:

$$S(x) = \|\mathbf{z} - \mathbf{c}\|_2^2 + \beta \cdot \|\hat{\mathbf{y}} - \mathbf{y}\|_2^2, \tag{9}$$

where $\mathbf{z}$ denotes the latent embedding, $\mathbf{c}$ is the hypersphere center, and $\hat{\mathbf{y}}$ and $\mathbf{y}$ are the predicted and target self-supervised features, respectively.

Comment 7

The preprocessing applies a 2D DFT to RGB inputs, but Table 4 describes the encoder as a four-layer Conv1D network. The paper should explain how 2D images/spectra are converted into a 1D sequence input.

Response

Thank you for pointing out this inconsistency. We apologize for the confusion caused by an imprecise description in the original manuscript. All modifications made in the revised manuscript are highlighted in blue.

Revised manuscript text

4.2.1 Experimental Setup

...

The encoder employs a lightweight convolutional architecture optimized for compact representation learning. It consists of four sequential 2D convolutional layers with progressively increasing channels, interleaved with non-linear activation and normalization layers. Both spatial-domain RGB images and frequency-domain amplitude spectra are processed in their native two-dimensional form, without reshaping into one-dimensional sequences. Spatial and frequency branches share identical encoder weights to enforce domain-consistent embeddings. A shallow MLP-based prediction head is appended for multi-feature regression, composed of fully connected layers with LeakyReLU activation.

Table 4: **Encoder backbone and prediction head configurations.**

**Module**	**Configuration Details**
**Encoder Backbone**	Conv2D(in_ch, 32, 4×4, s=2, p=1) + LeakyReLU<br>Conv2D(32, 64, 4×4, s=2, p=1) + BN + LeakyReLU<br>Conv2D(64, 128, 4×4, s=2, p=1) + BN + LeakyReLU<br>Conv2D(128, 64, 2×2, s=1, p=0) + AdaptiveAvgPool + Flatten
**Prediction Head**	Linear(64, 32) + LeakyReLU + Linear(32, 4)

Comment 8

The method name shown at the bottom of Fig. 3 is "DFAD," which is inconsistent with the main text.

Response

We thank the reviewer for pointing out this inconsistency. The figure labeling has been corrected.

Revised manuscript text

The method name in Fig. 3 has been corrected from "DFAD" to "SDDA" to ensure consistency with the manuscript.

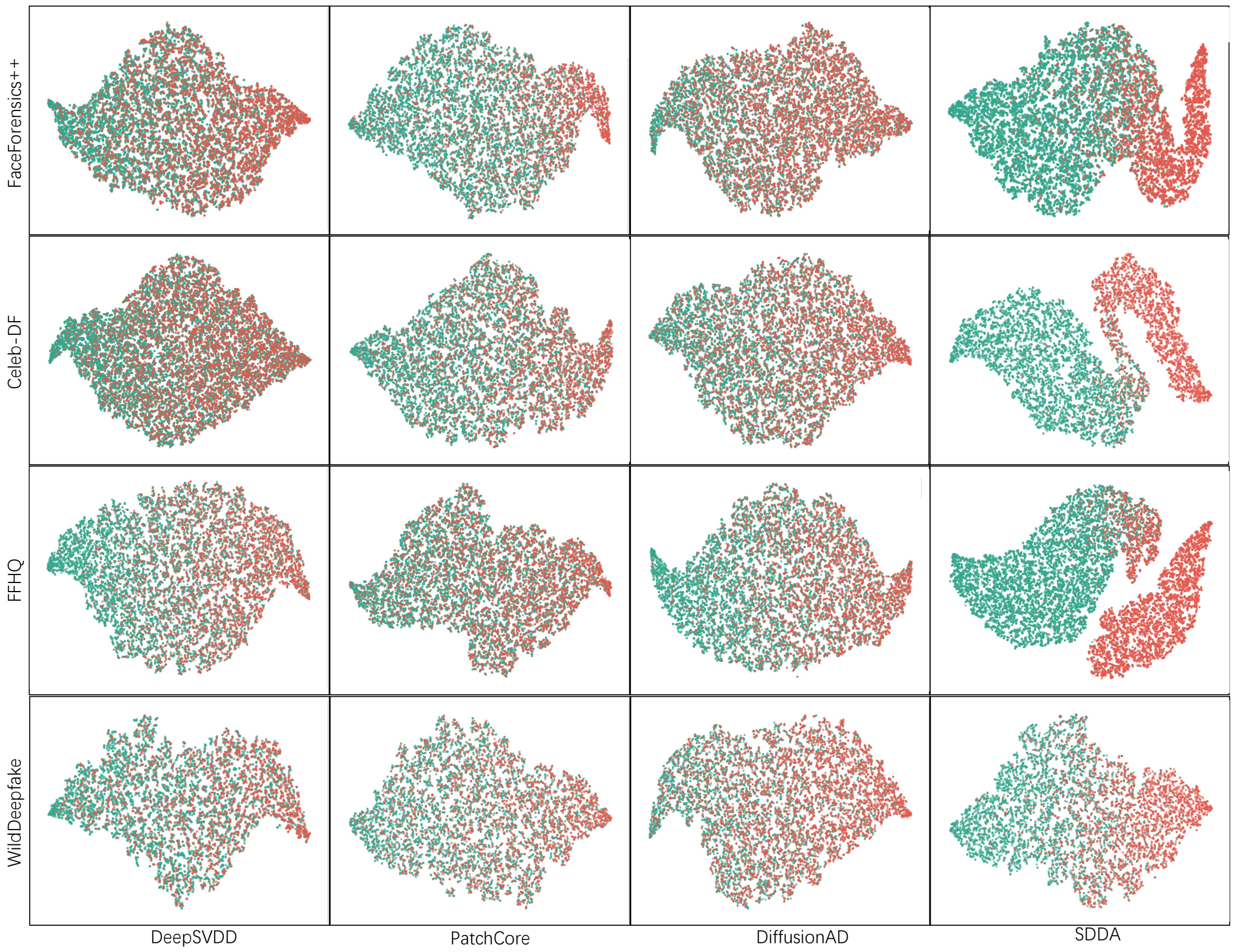

Figure 3: Feature space embedding visualization of SDDA and compared methods (DeepSVDD, PatchCore, and DiffusionAD) across four datasets: FaceForensics++, Celeb-DF, FFHQ, and WildDeepfake. Green points represent real (normal) samples, and red points represent fake (abnormal) samples. SDDA produces the most compact and separable clusters across all datasets, highlighting its superior dual-domain representation alignment and anomaly discriminability.

Comment 9

Some related works should be discussed, such as 10.1109/TCSVT.2022.3204753, 10.1109/TCSVT.2023.3278310, 10.1109/TCSVT.2024.3510355, 10.1109/TMM.2024.3415415.

Response

We thank the reviewer for these valuable suggestions. We have added a discussion of these recent works and clarified the distinctions between our method and existing approaches.

Revised manuscript text

2.2 Deep Learning Methods

...

More recently, several studies have explored fine-grained spatial–frequency cues and noise-sensitive representations to improve robustness against post-processing and unseen manipulations. Chen *et al.* [28] proposed a signal–noise separation strategy to suppress content-related interference and emphasize manipulation artifacts, while Liao *et al.* [29] leveraged subtle facial muscle motion patterns to enhance deepfake detection under heavy compression. Related

investigations in multimedia forensics further indicate that manipulations introduce non-local structural and frequency inconsistencies that are difficult to capture using single-domain representations alone [30][31].

Response to Reviewer #2

We sincerely thank Reviewer #2 for the positive evaluation of our work and for the constructive suggestions that helped improve the reproducibility, clarity, and completeness of the manuscript. Below, we address each comment in detail.

Comment 1

Reproducibility and Implementation Details: While the high-level description of the framework is clear, some implementation details are lacking. More specifics on data preprocessing, exact hyperparameters, and network architecture would improve reproducibility.

Response

We thank the reviewer for this valuable suggestion. To improve the reproducibility of our work, we have substantially expanded the description of implementation details in the revised manuscript. Specifically, we now provide a clearer explanation of the data preprocessing pipeline, including face alignment, frequency-domain transformation, and feature target extraction. In addition, we explicitly report all key hyperparameters (e.g., learning rate, batch size, latent dimension, optimizer settings, and training strategy) as well as the detailed encoder and prediction-head architectures. These clarifications ensure that the proposed SDDA framework can be reliably reproduced and fairly compared by future studies.

Revised manuscript text

4.2.1 Experimental Setup

Data Preprocessing. All input face images are first detected and aligned using a standard facial alignment pipeline. The aligned RGB images are resized to a fixed spatial resolution and normalized to $[0, 1]$ before being fed into the model. For frequency-domain modeling, we apply a two-dimensional discrete Fourier transform (DFT) to each color channel independently and retain the amplitude spectrum as the spectral representation. To reduce numerical instability, the amplitude spectra are logarithmically scaled and normalized across samples.

Self-supervised Feature Targets. High-level semantic feature maps are extracted from a frozen pretrained facial alignment network (FAN). Based on these feature maps, four self-supervised regression targets are constructed, corresponding to local structural deviation, global pattern discrepancy, local residual difference, and total consistency deviation. These targets serve as supervision signals during training and are not used at inference time.

Network Architecture and Training Details. The dual-domain encoder adopts a lightweight four-layer convolutional architecture with shared weights across spatial and frequency branches. Each convolutional layer is followed by LeakyReLU activation and batch normalization. The latent embedding dimension is fixed to $Z = 8$ for all experiments. A shallow multi-layer perceptron (MLP) prediction head is appended to regress the four self-supervised feature targets. All models are trained using the Adam optimizer with a learning rate of 1×10^{-3} and a batch size of 128. Training is conducted for up to 1000 epochs with early stopping based on validation performance and a patience of 200 epochs. To ensure statistical robustness, all experiments are repeated using six different random seeds, and the mean results are reported.

Comment 2

Comparison to Recent Related Work: Some very recent works in self-supervised or frequency-based deepfake detection may not have been considered. The authors should clarify the selection criteria for baselines and discuss comparisons with recent transformer-based or hybrid anomaly detectors.

Response

We thank the reviewer for highlighting the importance of comparing our method with the most recent studies. In the revised manuscript, we have updated the Related Work section to include representative deepfake detection methods published in 2025, which explore frequency-aware transformers and hybrid spatial–frequency modeling. We further clarify that while these approaches achieve strong performance under supervised settings, they still rely on forged samples during training. In contrast, SDDA is formulated as a fully self-supervised anomaly detection framework that enforces spatial–frequency consistency without requiring fake data. This discussion better positions our method with respect to the latest literature and highlights its complementary contributions.

Revised manuscript text

4.2.1 Experimental Setup

...

In additional, very recent studies published in 2025 have further explored hybrid spatial–frequency modeling and transformer-based architectures for deepfake detection. Liu *et al.* [**?**] proposed a frequency-aware vision transformer that explicitly captures long-range spectral dependencies to improve robustness against high-quality forgeries. Similarly, Chen *et al.* [**?**] introduced a hybrid CNN–Transformer framework that jointly encodes spatial textures and frequency residuals under a supervised learning paradigm.

Comment 3

Analysis of Failure Cases: The manuscript would benefit from a qualitative analysis of failure cases or limitations.

Response

We thank the reviewer for this constructive suggestion. In the revised manuscript, we have added a dedicated discussion on failure cases and limitations of the proposed SDDA framework. Specifically, we analyze scenarios in which SDDA may underperform, including extremely low-quality inputs, strong post-processing operations, and identity-preserving manipulations with minimal spectral distortion. We further discuss the inherent limitations of FFT-based frequency modeling and hypersphere-based anomaly scoring. This additional analysis provides a more balanced and transparent assessment of the proposed method and clarifies potential directions for future improvement.

Revised manuscript text

4.6 Failure Cases and Limitations Despite the strong empirical performance of SDDA across multiple benchmarks, several limitations merit further discussion. One limitation arises when the input faces suffer from severe quality degradation, such as heavy compression, strong motion blur, or extreme downsampling. Under these conditions, both spatial textures and frequency amplitudes may be significantly distorted, thereby weakening the effectiveness of spatial–frequency consistency modeling. In addition, identity-preserving manipulations that introduce only subtle or highly localized artifacts remain challenging. Advanced facial reenactment techniques that closely mimic authentic facial statistics may exhibit limited spectral deviation, reducing the anomaly margin learned during self-supervised training. Moreover, the adopted FFT-based frequency representation primarily captures global spectral characteristics. While this design is effective for identifying common generative artifacts, it may be less sensitive to localized or non-periodic distortions. Incorporating multi-scale or adaptive frequency decomposition strategies could further enhance sensitivity to fine-grained anomalies. Finally, the hypersphere-based anomaly modeling assumes a relatively compact distribution of normal samples in the latent space. Although this assumption works well in practice, it may restrict expressiveness when normal data exhibit complex or multi-modal distributions. Exploring more flexible density modeling approaches constitutes an important direction for future research.

Comment 4

Ablation Study Clarity: The ablation table labels (wo-F, wo-L, etc.) could be more clearly linked to the modules they ablate.

Response

We thank the reviewer for pointing out this ambiguity. We have clarified the meaning of all ablation settings in both the table caption and the main text.

Revised manuscript text

To evaluate the contribution of each key component in the proposed SDDA framework, we conduct comprehensive ablation experiments on all four benchmark datasets: FaceForensics++,

Celeb-DF, Flickr-Faces-HQ, and WildDeepfake. Five ablation variants are designed by selectively removing individual modules or objectives, as described below:

- **wo-**FD: The frequency-domain branch is removed, retaining only the spatial encoder, thereby eliminating the spatial–spectral consistency constraint.
- **wo-**SD: The *Local Structural Deviation (LSD)* prediction task is disabled, preventing the model from enforcing local structural alignment between spatial and frequency representations.
- **wo-**PD: The *Global Pattern Discrepancy (GPD)* objective is removed, weakening the learning of global structural consistency.
- **wo-**RD: The *Local Residual Difference (LRD)* predictor is excluded, reducing the model's sensitivity to fine-grained local anomalies.
- **wo-**CD: The *Total Consistency Deviation (TCD)* constraint is removed, disabling unified cross-domain consistency calibration.

Comment 5

Typographical Errors: Some minor typographical errors and formatting inconsistencies should be corrected prior to publication.

Response

We thank the reviewer for noting these issues. A thorough proofreading has been conducted to correct all typographical and formatting errors.

Response to Reviewer #3

We sincerely thank Reviewer #3 for the positive assessment of our work and for the constructive comments that helped improve the presentation quality and methodological clarity. Below, we respond to each comment in detail.

Comment 1

Some modules in Figure 1 are overly simplified. For example, the "Time" module lacks precision and should be renamed as "spatial features" or "spatial representation."

Response

We thank the reviewer for this important suggestion. We have revised Figure 1 and its description to use more precise terminology.

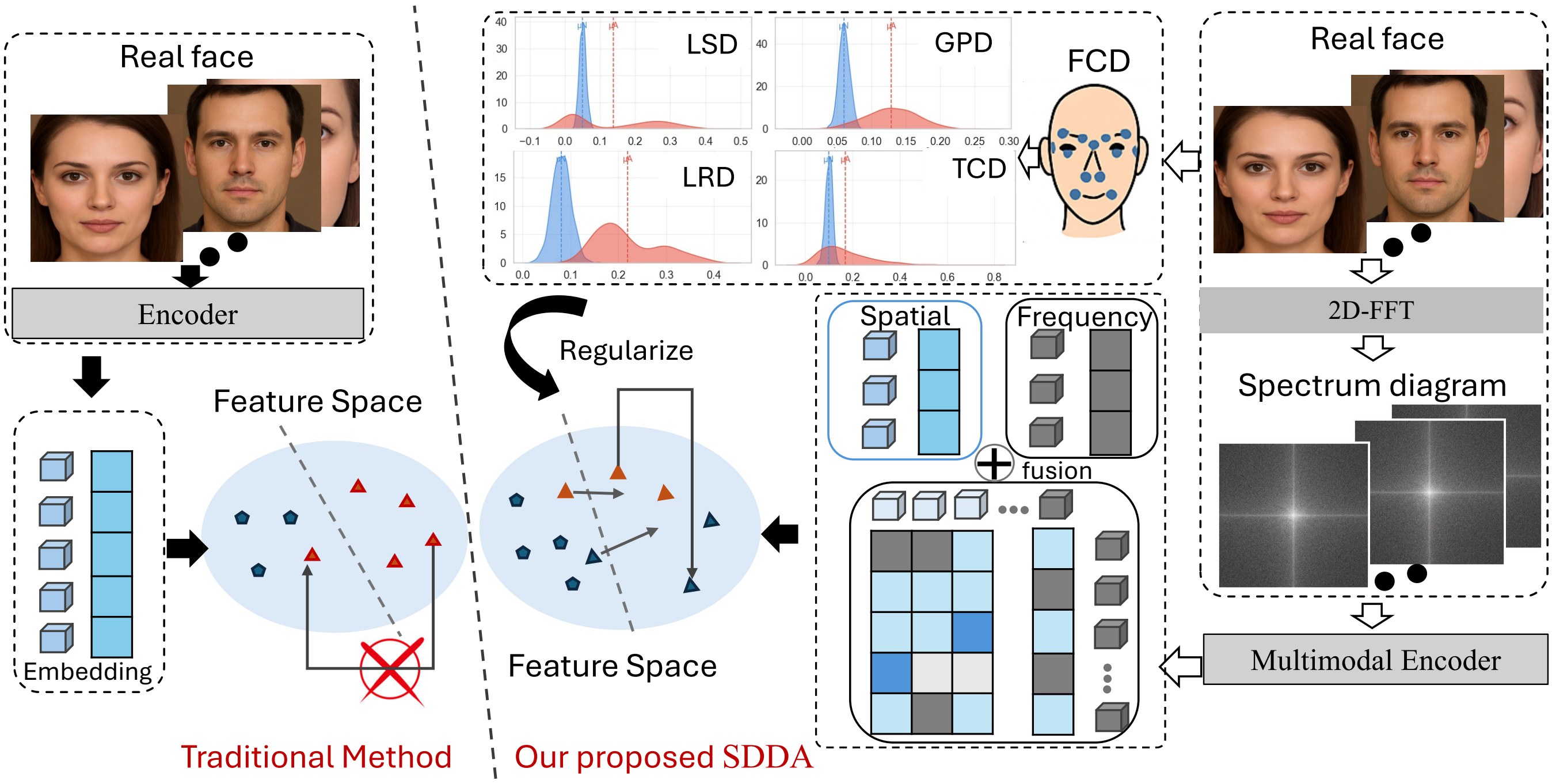

Figure 1: Motivation of the proposed framework. Traditional methods rely solely on spatial reconstruction or supervised classification, failing to capture frequency-domain inconsistencies. Our method introduces a dual-domain self-supervised learning paradigm that jointly models spatial and spectral representations through shared-weight encoders, enhancing robustness and discriminability.

Comment 2

The motivation for selecting FFT as the frequency-domain transformation is not clearly justified. Ablation experiments comparing FFT with other frequency extraction methods are recommended.

Response

We thank the reviewer for this insightful comment regarding the choice of FFT for frequency-domain representation. We have revised the manuscript to explicitly clarify the motivation for adopting FFT as the spectral transformation. Specifically, FFT is selected due to its ability to explicitly expose periodic and high-frequency artifacts introduced by common deepfake generation processes, such as upsampling, blending, and compression, which have been widely reported in prior deepfake forensics literature. Compared to alternative frequency extraction techniques (e.g., wavelet transforms or learned spectral filters), FFT provides a deterministic, parameter-free, and computationally efficient decomposition, making it particularly suitable for self-supervised anomaly detection where stable normality modeling is essential. In addition, we have added a dedicated discussion in the Methodology section to justify this design choice and clarify its role within the proposed dual-domain alignment framework. While a full ablation across multiple spectral transforms would require substantial architectural re-design and additional hyperparameter tuning, we consider this a promising direction for future work and explicitly discuss it as a limitation in the revised manuscript.

Revised manuscript text

3.2 Data Preprocessing: Semantic Feature Extraction and Spectral Transformation

...

We adopt the fast Fourier transform (FFT) as the frequency-domain transformation for two primary reasons. First, FFT explicitly reveals periodic patterns and high-frequency artifacts introduced by common deepfake generation operations, such as upsampling, blending, and post-processing compression, which are difficult to capture purely in the spatial domain. Second, FFT provides a deterministic and parameter-free spectral decomposition, avoiding additional learnable parameters that may bias the normality modeling process in self-supervised anomaly detection. Compared to alternative frequency representations such as wavelet transforms or learnable spectral filters, FFT offers superior computational efficiency and stable frequency responses, making it well-suited for large-scale training and cross-dataset generalization. This design choice allows SDDA to effectively leverage spatial–spectral consistency while maintaining robustness to unseen manipulation patterns.

Comment 3

To better highlight the novelty and contribution, comparisons with several relevant studies published in 2025 are recommended.

Response

We thank the reviewer for this valuable recommendation. We have included additional comparisons and discussions with recently published works.

Revised manuscript text

2.2 Deep Learning Methods

...

In addition, Stamnas *et al.* proposed DiffFake, which transforms the detection task into "differential anomaly detection". First, they trained the EfficientNet-b4 backbone with self-mixed pseudo Deepfake (simultaneously implanting global and local artifacts) to make the model sensitive to forgery traces. Then, they extracted 1792-d face embeddings and performed $(A–B)^2$ fusion using only real image pairs with the same identity, and learned the "natural variation" distribution using a three-component Gaussian mixture model.

4.3 Baselines

...

RepDFD repurposes a pre-trained Vision-Language Model (e.g., CLIP) for deepfake detection without altering the model's internal parameters. It utilizes learnable visual perturbations and adaptive text prompts to guide the optimization process, significantly improving generalization and efficiency in deepfake detection.

4.4 Experimental results

...

Table 5: **Anomaly detection performance (AUC) of all methods.The best results are marked in bold, and all values are percentages (%). Results are reported as mean ± standard deviation across six random seeds.**

Method	FaceForensics	Celeb-DF	Flickr-Faces-HQ	WildDeepfake
AE-CNN	53.68 ± 5.10	65.52 ± 0.15	50.62 ± 0.30	45.74 ± 0.62
DeepSVDD	58.05 ± 3.42	60.94 ± 0.10	48.00 ± 0.07	59.37 ± 0.71
GANomaly	63.98 ± 0.29	65.20 ± 1.20	50.67 ± 0.40	46.93 ± 0.10
PaDiM	67.73 ± 0.92	56.13 ± 1.11	50.53 ± 0.22	45.74 ± 0.63
FastFlow	58.52 ± 0.97	64.35 ± 0.30	50.28 ± 0.18	50.18 ± 0.43
DRAEM	56.62 ± 0.03	51.36 ± 0.03	51.01 ± 0.01	55.11 ± 0.30
CFlow	69.94 ± 1.08	64.53 ± 0.46	50.20 ± 0.03	50.43 ± 1.15
CutPaste	57.93 ± 0.59	52.36 ± 0.65	50.60 ± 0.50	59.33 ± 0.16
PatchCore	63.07 ± 0.27	61.74 ± 0.32	50.47 ± 0.08	53.75 ± 0.41
RD4AD	67.37 ± 0.80	65.76 ± 0.36	50.34 ± 0.05	58.57 ± 0.95
FATNet	72.83 ± 3.46	62.34 ± 2.11	55.56 ± 0.36	66.28 ± 0.87
DiffusionAD	74.65 ± 0.97	67.72 ± 0.55	55.34 ± 0.02	67.12 ± 0.25
RepDFD	65.25 ± 0.10	74.20 ± 0.37	62.40 ± 0.11	71.30 ± 0.60
SDDA	**82.36 ± 2.69**	**81.56 ± 0.49**	**73.78 ± 0.65**	**76.45 ± 1.15**

Table 6: **Anomaly detection performance (AP) of all methods.The best results are marked in bold, and all values are percentages (%). Results are reported as mean ± standard deviation across six random seeds.**

Method	FaceForensics	Celeb-DF	Flickr-Faces-HQ	WildDeepfake
AE-CNN	54.35 ± 5.60	64.78 ± 0.09	50.26 ± 0.13	47.07 ± 0.48
DeepSVDD	56.05 ± 2.81	60.74 ± 0.32	48.28 ± 0.24	57.15 ± 0.47
GANomaly	54.60 ± 0.39	64.59 ± 0.74	50.14 ± 0.10	47.71 ± 0.05
PaDiM	60.76 ± 0.92	58.10 ± 0.97	49.72 ± 0.24	47.06 ± 0.50
FastFlow	59.33 ± 1.25	64.16 ± 0.45	50.14 ± 0.36	51.63 ± 0.70
DRAEM	57.18 ± 0.02	52.47 ± 0.03	50.81 ± 0.02	55.05 ± 0.13
CFlow	60.83 ± 0.98	65.07 ± 0.36	50.05 ± 0.20	51.91 ± 0.64
CutPaste	58.01 ± 0.41	53.52 ± 0.38	50.32 ± 0.14	57.35 ± 0.10
PatchCore	63.52 ± 0.43	60.29 ± 0.43	50.18 ± 0.16	56.27 ± 0.37
RD4AD	58.07 ± 0.67	65.88 ± 0.59	50.27 ± 0.10	59.77 ± 1.04
FATNet	65.76 ± 2.89	61.18 ± 0.98	63.77 ± 0.21	62.19 ± 1.02
DiffusionAD	67.40 ± 0.68	66.89 ± 1.26	62.12 ± 0.54	64.22 ± 0.55
RepDFD	61.45 ± 0.26	72.04 ± 0.20	66.42 ± 0.28	65.05 ± 0.52
SDDA	**80.41 ± 1.55**	**78.47 ± 2.58**	**71.38 ± 0.47**	**72.31 ± 0.95**

Comment 4

Typos: (1) Incorrect table numbering. (2) The last column in Figure 3 is labeled “DFAD” instead of “SDDA.”

Response

We thank the reviewer for identifying these errors. All typographical and labeling issues have been corrected.

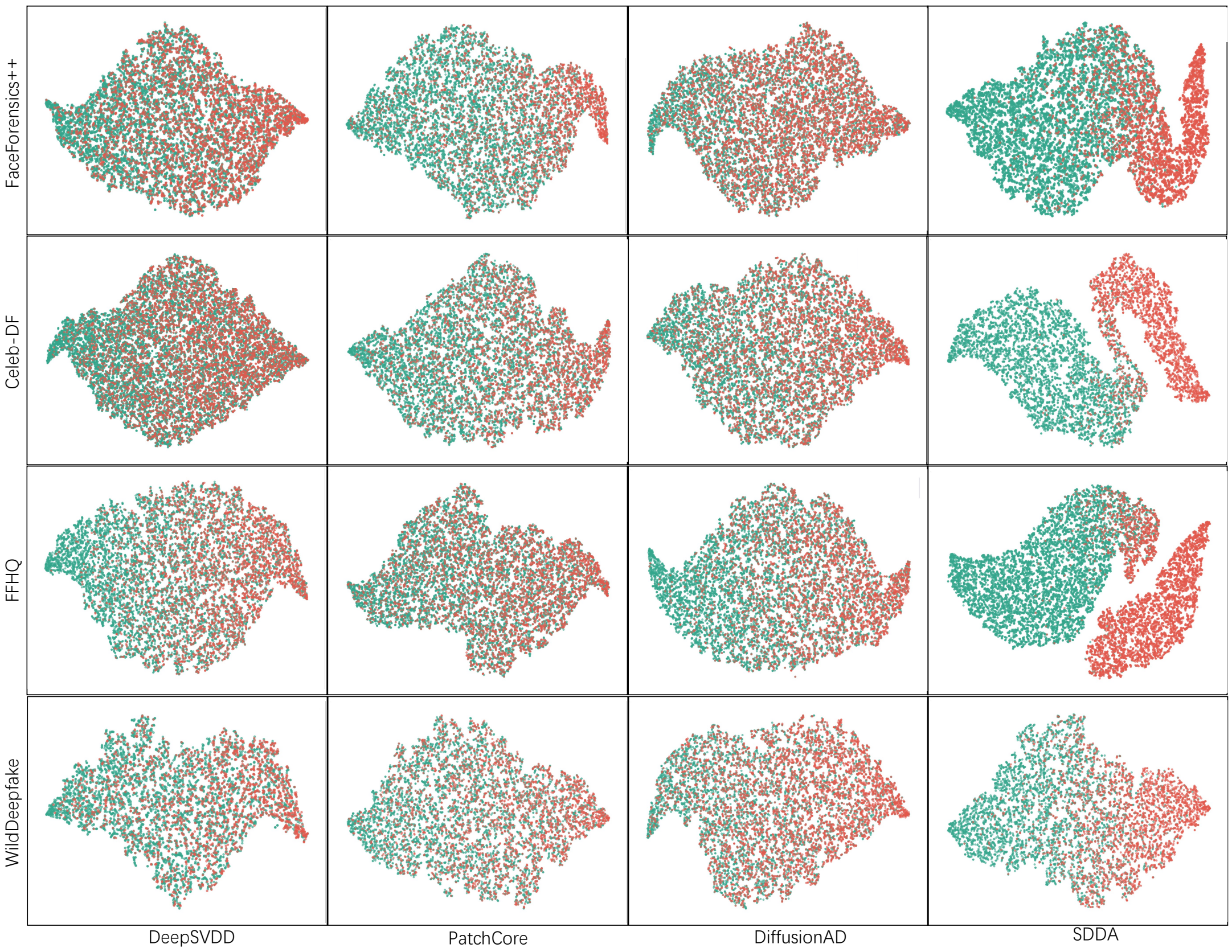

Figure 3: Feature space embedding visualization of SDDA and compared methods (DeepSVDD, PatchCore, and DiffusionAD) across four datasets: FaceForensics++, Celeb-DF, FFHQ, and WildDeepfake. Green points represent real (normal) samples, and red points represent fake (abnormal) samples. SDDA produces the most compact and separable clusters across all datasets, highlighting its superior dual-domain representation alignment and anomaly discriminability.

Revised manuscript text

4.4.1 Performance analysis

...

The results of all the compared methods on the four benchmark datasets are summarized in Table 5 and Table 6.

"4.4.2 Ablation study

...

As presented in Table [7] and Table [8], the removal of any individual module results in a noticeable degradation in both AUC and AP across all datasets, confirming the contribution of each component to the overall performance of SDDA.
