# Supplementary material for: Dual-domain self-supervised feature alignment via spectral–spatial representation learning for deepfake anomaly detection
Source: PLoS One. 2026 Sep 11;21(9):e0358049. doi: 10.1371/journal.pone.0358049 (PMC13568503; doi:10.1371/journal.pone.0358049)
Supplement: Supplementary file 2 [file pone.0358049.s003.pdf]

Response to Reviewers

Manuscript ID: PONE-D-25-63428

Title: *Dual-Domain Self-Supervised Feature Alignment via Spectral–Spatial Representation Learning for Deepfake Anomaly Detection*

Response to the Academic Editor

We sincerely thank the Academic Editor for the careful handling of our manuscript and for providing us with the opportunity to revise our work.

We have carefully addressed all comments from the reviewers and revised the manuscript accordingly. The revisions mainly focus on improving the experimental design, clarifying the methodology, enhancing reproducibility, and strengthening the overall presentation of the paper.

All changes have been clearly marked in the revised manuscript with track changes. Detailed point-by-point responses to the reviewers' comments are provided in the accompanying "Response to Reviewers" document. We hope that the revised manuscript meets the journal's requirements, and we appreciate your consideration.

Reviewer 4

Reviewer Comment:

Overall, the paper presents an interesting attempt to integrate spatial and frequency-domain representations for deepfake anomaly detection. However, several methodological clarifications and experimental improvements are needed to better demonstrate the novelty and robustness of the proposed approach.

Reply: We sincerely thank the reviewer for the positive evaluation of our work and for recognizing the potential of integrating spatial and frequency-domain representations for deepfake anomaly detection.

We appreciate the reviewer's insightful comments regarding the need for clearer methodological descriptions and more rigorous experimental validation. In this revision, we have carefully addressed these concerns by substantially improving the clarity of the proposed method, expanding the experimental design, and strengthening the evaluation protocols. In particular, we have introduced additional experiments to better assess the robustness and generalization ability of our approach, and we have provided more detailed explanations of key components to enhance reproducibility.

We believe that these revisions have significantly improved the quality of the manuscript and have better demonstrated the novelty and effectiveness of the proposed framework. Detailed responses to each specific comment are provided below.

Reviewer Comment 1: Dataset selection

The evaluation relies on four datasets: FaceForensics++, Celeb-DF, Flickr-Faces-HQ, and WildDeepfake. Most of these datasets were introduced before 2020. Considering the rapid progress in deepfake generation techniques, it would strengthen the paper to include evaluations on more recent datasets or provide justification for relying solely on older benchmarks.

Reply: Thank you for this valuable suggestion. We agree that evaluating on more recent and challenging datasets is important, given the rapid advances in deepfake generation techniques in recent years. To address this concern, we have expanded our experimental evaluation by incorporating the ForgeryNet dataset, a large-scale and more recent benchmark that includes a diverse set of forgery types, post-processing perturbations, and comprehensive annotations. This addition allows us to further assess the robustness and generalization capability of the proposed method under more realistic and challenging conditions. The specific revisions are shown below.

4 Experiments

The general training process of our proposed SDDA model is shown in Table 2.

...

4.1 Datasets

...

Brief information about the datasets used in this study is shown in Table 3.

...

ForgeryNet: ForgeryNet is a large-scale face forgery benchmark containing 1.44 million real images, 1.46 million fake images, and over 220,000 videos from more than 5,400 identities. It includes 15 manipulation methods across face swap, face transfer, face reenactment, and face editing, with 36 perturbation types to simulate real-world distortions. The dataset provides annotations for image and video forgery classification, as well as spatial and temporal forgery localization. Considering the large scale of the dataset and computational constraints, we construct a representative subset for evaluation. Specifically, we utilize the image subset and sample frames from videos at a fixed interval (one frame every 10 frames) to reduce redundancy while preserving data diversity. We further ensure a balanced distribution across real and fake samples as well as different manipulation categories. The final subset contains approximately 50,000 real images and 50,000 fake images, covering all major manipulation types in ForgeryNet. This strategy enables efficient training and evaluation while maintaining the variability of manipulation types and visual conditions.

4.2 Experimental Design and Evaluation Metrics

...

4.2.1 Experimental Setup

...

The specific configuration parameters of the encoder backbone and prediction head are shown in Table 4.

Table 1: **Statistics of the Deepfake datasets used in this study.**

**Datasets**		**Specifications**		
Type	Dataset	Format	Resolution	Count
Video-based	FaceForensics++	MP4	720p	1,977 videos / 1,000 sequences
Video-based	Celeb-DF	MP4	256p–720p	5,639 videos
Image-based	FFHQ	PNG	1024×1024	70,000 images
Video-based	WildDeepfake	MP4	480p–1080p	7,314 sequences
Video-based	ForgeryNet	MP4 / JPG	240p–1080p	221,247 videos + 2.9M images

4.4 Experimental results

...

4.4.1 Performance analysis

...

Table 2: **Anomaly detection performance (AUC) of all methods.The best results are marked in bold, and all values are percentages (%). Results are reported as mean ± standard deviation across six random seeds.**

Method	FaceForensics	Celeb-DF	Flickr-Faces-HQ	WildDeepfake	ForgeryNet
AE-CNN	53.68 ± 5.10	65.52 ± 0.15	50.62 ± 0.30	45.74 ± 0.62	67.84 ± 2.21
DeepSVDD	58.05 ± 3.42	60.94 ± 0.10	48.00 ± 0.07	59.37 ± 0.71	74.21 ± 0.20
GANomaly	63.98 ± 0.29	65.20 ± 1.20	50.67 ± 0.40	46.93 ± 0.10	69.11 ± 1.42
PaDiM	67.73 ± 0.92	56.13 ± 1.11	50.53 ± 0.22	45.74 ± 0.63	71.43 ± 1.71
FastFlow	58.52 ± 0.97	64.35 ± 0.30	50.28 ± 0.18	50.18 ± 0.43	75.04 ± 0.18
DRAEM	56.62 ± 0.03	51.36 ± 0.03	51.01 ± 0.01	55.11 ± 0.30	68.37 ± 2.15
CFlow	69.94 ± 1.08	64.53 ± 0.46	50.20 ± 0.03	50.43 ± 1.15	80.86 ± 0.11
CutPaste	57.93 ± 0.59	52.36 ± 0.65	50.60 ± 0.50	59.33 ± 0.16	73.56 ± 0.16
PatchCore	63.07 ± 0.27	61.74 ± 0.32	50.47 ± 0.08	53.75 ± 0.41	70.26 ± 2.19
RD4AD	67.37 ± 0.80	65.76 ± 0.36	50.34 ± 0.05	58.57 ± 0.95	62.08 ± 1.22
FATNet	72.83 ± 3.46	62.34 ± 2.11	55.56 ± 0.36	66.28 ± 0.87	81.44 ± 1.53
DiffusionAD	74.65 ± 0.97	67.72 ± 0.55	55.34 ± 0.02	67.12 ± 0.25	65.92 ± 2.70
RepDFD	65.25 ± 0.10	74.20 ± 0.37	62.40 ± 0.11	71.30 ± 0.60	70.42 ± 2.33
SDDA	**82.36 ± 2.69**	**81.56 ± 0.49**	**73.78 ± 0.65**	**76.45 ± 1.15**	**83.13 ± 1.12**

4.4.2 Ablation study

...

4.4.4 Visualization

Reviewer Comment 2: Evaluation protocol

Reviewer Comment:

The training set contains only authentic samples, while both the validation and test sets include real and fake samples. However, it appears that the same manipulation methods are present in both validation and test sets. This may expose the model to the anomaly distribution during model selection. It would be helpful to clarify this setting and consider cross-manipulation evaluation to better demonstrate generalization to unseen forgeries.

Reply: Thank you for this valuable comment. In our experimental setup, the training set consists exclusively of authentic samples, whereas both the validation and test sets contain real and manipulated samples. We acknowledge that using the same manipulation methods in the validation and test sets may expose the model selection process to the target anomaly distribution. To provide a more rigorous assessment of generalization, we conducted additional cross-dataset experiments in which the model was trained on one dataset and evaluated on other datasets containing previously unseen manipulation methods. The results show that

Table 3: **Anomaly detection performance (AP) of all methods.The best results are marked in bold, and all values are percentages (%). Results are reported as mean ± standard deviation across six random seeds.**

Method	FaceForensics	Celeb-DF	Flickr-Faces-HQ	WildDeepfake	ForgeryNet
AE-CNN	54.35 ± 5.60	64.78 ± 0.09	50.26 ± 0.13	47.07 ± 0.48	63.26 ± 0.16
DeepSVDD	56.05 ± 2.81	60.74 ± 0.32	48.28 ± 0.24	57.15 ± 0.47	70.54 ± 2.50
GANomaly	54.60 ± 0.39	64.59 ± 0.74	50.14 ± 0.10	47.71 ± 0.05	62.45 ± 4.11
PaDiM	60.76 ± 0.92	58.10 ± 0.97	49.72 ± 0.24	47.06 ± 0.50	70.23 ± 2.40
FastFlow	59.33 ± 1.25	64.16 ± 0.45	50.14 ± 0.36	51.63 ± 0.70	71.20 ± 0.42
DRAEM	57.18 ± 0.02	52.47 ± 0.03	50.81 ± 0.02	55.05 ± 0.13	66.23 ± 0.46
CFlow	60.83 ± 0.98	65.07 ± 0.36	50.05 ± 0.20	51.91 ± 0.64	78.33 ± 0.23
CutPaste	58.01 ± 0.41	53.52 ± 0.38	50.32 ± 0.14	57.35 ± 0.10	74.57 ± 0.52
PatchCore	63.52 ± 0.43	60.29 ± 0.43	50.18 ± 0.16	56.27 ± 0.37	70.10 ± 0.30
RD4AD	58.07 ± 0.67	65.88 ± 0.59	50.27 ± 0.10	59.77 ± 1.04	59.41 ± 0.32
FATNet	65.76 ± 2.89	61.18 ± 0.98	63.77 ± 0.21	62.19 ± 1.02	77.60 ± 0.47
DiffusionAD	67.40 ± 0.68	66.89 ± 1.26	62.12 ± 0.54	64.22 ± 0.55	60.94 ± 1.35
RepDFD	61.45 ± 0.26	72.04 ± 0.20	66.42 ± 0.28	65.05 ± 0.52	72.51 ± 0.89
SDDA	**80.41 ± 1.55**	**78.47 ± 2.58**	**71.38 ± 0.47**	**72.31 ± 0.95**	**82.40 ± 1.37**

Table 4: **Ablation study of the contribution of each module in SDDA (AUC).The best results are marked in bold, and all values are percentages (%). Results are reported as mean ± standard deviation across six random seeds.**

Variant	FaceForensics	Celeb-DF	Flickr-Faces-HQ	WildDeepfake	ForgeryNet
wo-F	73.86 ± 1.45	69.84 ± 1.14	59.02 ± 0.65	60.12 ± 0.81	70.40 ± 0.23
wo-L	75.34 ± 2.03	70.35 ± 0.95	60.55 ± 0.49	61.24 ± 0.97	66.54 ± 0.54
wo-G	76.12 ± 1.78	70.78 ± 0.77	61.27 ± 0.58	62.05 ± 0.72	73.72 ± 0.40
wo-R	77.24 ± 1.56	71.34 ± 0.89	62.03 ± 0.64	63.22 ± 0.78	75.26 ± 1.15
wo-T	78.32 ± 1.34	72.45 ± 0.92	62.84 ± 0.56	65.17 ± 0.75	70.23 ± 0.69
**SDDA**	**82.36 ± 2.69**	**81.56 ± 0.49**	**73.78 ± 0.65**	**76.45 ± 1.15**	**83.13 ± 1.12**

SDDA consistently outperforms the competing methods, further demonstrating its strong robustness and generalization capability to unseen forgeries. The specific revisions are shown below.

4.4 Experimental results

4.4.3 Generalization Experiments

In this section, we evaluate the generalization performance of the SDDA model. We used data from the ForgeryNet dataset as the training dataset and the FaceForensics and Celeb-DF datasets as the test datasets. Similarly, we used data from the Flickr-Faces-HQ dataset as the training dataset and the WildDeepfake dataset as the test dataset. The results of the generalization experiments are shown in the table below.

The results show that the SDDA model exhibits superior generalization performance compared to the

Table 5: **Ablation study of the contribution of each module in SDDA (AP).The best results are marked in bold, and all values are percentages (%). Results are reported as mean ± standard deviation across six random seeds.**

Variant	FaceForensics	Celeb-DF	Flickr-Faces-HQ	WildDeepfake	ForgeryNet
wo-F	70.22 ± 1.31	66.05 ± 0.93	57.28 ± 0.52	58.03 ± 0.63	71.13 ± 1.05
wo-SD	72.09 ± 1.47	67.12 ± 0.88	58.61 ± 0.74	59.10 ± 0.69	74.20 ± 0.41
wo-PD	73.01 ± 1.30	68.22 ± 0.65	59.33 ± 0.63	60.21 ± 0.55	70.50 ± 1.27
wo-RD	74.12 ± 1.25	68.85 ± 0.82	60.41 ± 0.59	60.84 ± 0.62	76.73 ± 0.42
wo-TD	75.65 ± 1.17	69.54 ± 0.77	60.96 ± 0.68	61.83 ± 0.58	73.51 ± 1.22
**SDDA**	**80.41 ± 1.55**	**78.47 ± 2.58**	**71.38 ± 0.47**	**72.31 ± 0.95**	**82.40 ± 1.37**

models listed. Overall, when trained on the ForgeryNet dataset and tested on the Celeb-DF dataset, the SDDA model maintains good performance, outperforming other comparative models. However, when trained on the Flickr-Faces-HQ dataset and tested on the WildDeepfake dataset, the performance of the SDDA model decreases compared to training and testing directly on the WildDeepfake dataset. Nevertheless, this model still outperforms other comparative models. We speculate that the performance degradation is due to significant data differences between the Flickr-Faces-HQ dataset, which primarily uses images, and the WildDeepfake dataset, which processes videos into images.

Table 6: **Anomaly detection performance in generalization experiments. All values are percentages(%) and the best results are marked in bold.**

Methods	ForgeryNet → FaceForensics		ForgeryNet → Celeb-DF		Flickr-Faces-HQ → WildDeepfake	
	AP	AUC	AP	AUC	AP	AUC
DeepSVDD	60.20 ± 3.22	59.45 ± 2.24	64.27 ± 0.23	61.44 ± 0.50	67.20 ± 0.48	65.15 ± 0.29
CFlow	67.05 ± 1.23	58.91 ± 0.17	62.99 ± 0.26	63.24 ± 0.16	50.33 ± 0.98	51.47 ± 0.20
CutPaste	60.37 ± 0.53	59.33 ± 0.26	54.20 ± 1.73	51.42 ± 1.45	54.21 ± 0.61	53.62 ± 1.32
FATNet	73.38 ± 2.68	66.50 ± 1.78	63.71 ± 1.27	59.97 ± 1.17	64.25 ± 0.15	65.31 ± 0.58
RepDFD	69.70 ± 0.25	65.47 ± 0.75	72.35 ± 2.56	71.08 ± 0.43	68.18 ± 0.41	67.62 ± 1.57
**SDDA**	**75.15 ± 2.20**	**76.56 ± 0.62**	**75.47 ± 0.32**	**74.07 ± 1.46**	**70.26 ± 0.97**	**68.17 ± 0.83**

Reviewer Comment 3: Related work coverage

Reviewer Comment:

The related work section introduces only a limited number of anomaly detection–based deepfake detection approaches. Given the growing literature in this direction, the authors may consider adding a dedicated subsection summarizing existing anomaly detection methods to provide a more comprehensive overview.
Reply: Thank you for this valuable suggestion. We agree that anomaly detection has become an increasingly important direction in deepfake detection, especially for addressing the limitations of supervised classifiers in open-set scenarios. To provide a more comprehensive overview of this research line, we have added a dedicated subsection in the Related Work section entitled "Anomaly Detection-Based Deepfake Detection

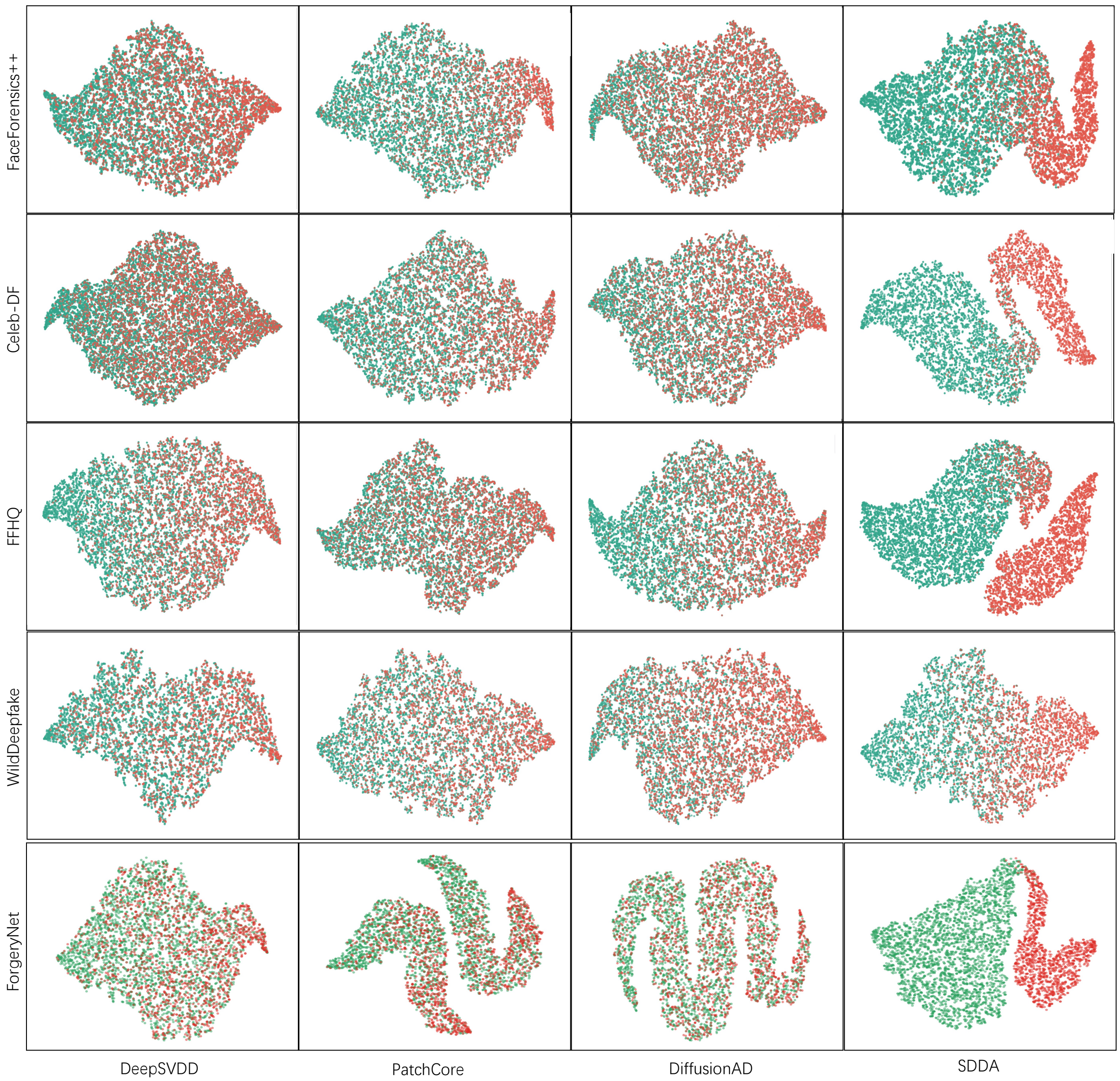

Figure 1: Feature space embedding visualization of SDDA and compared methods (DeepSVDD, PatchCore, and DiffusionAD) across five datasets: FaceForensics++, Celeb-DF, FFHQ, WildDeepfake, and ForgeryNet. Green points represent real (normal) samples, and red points represent fake (abnormal) samples. SDDA produces the most compact and separable clusters across all datasets, highlighting its superior dual-domain representation alignment and anomaly discriminability.

Methods."

In this new subsection, we summarize representative approaches that model authentic facial distributions and identify manipulated samples as outliers. Specifically, we discuss reconstruction-based methods, one-class

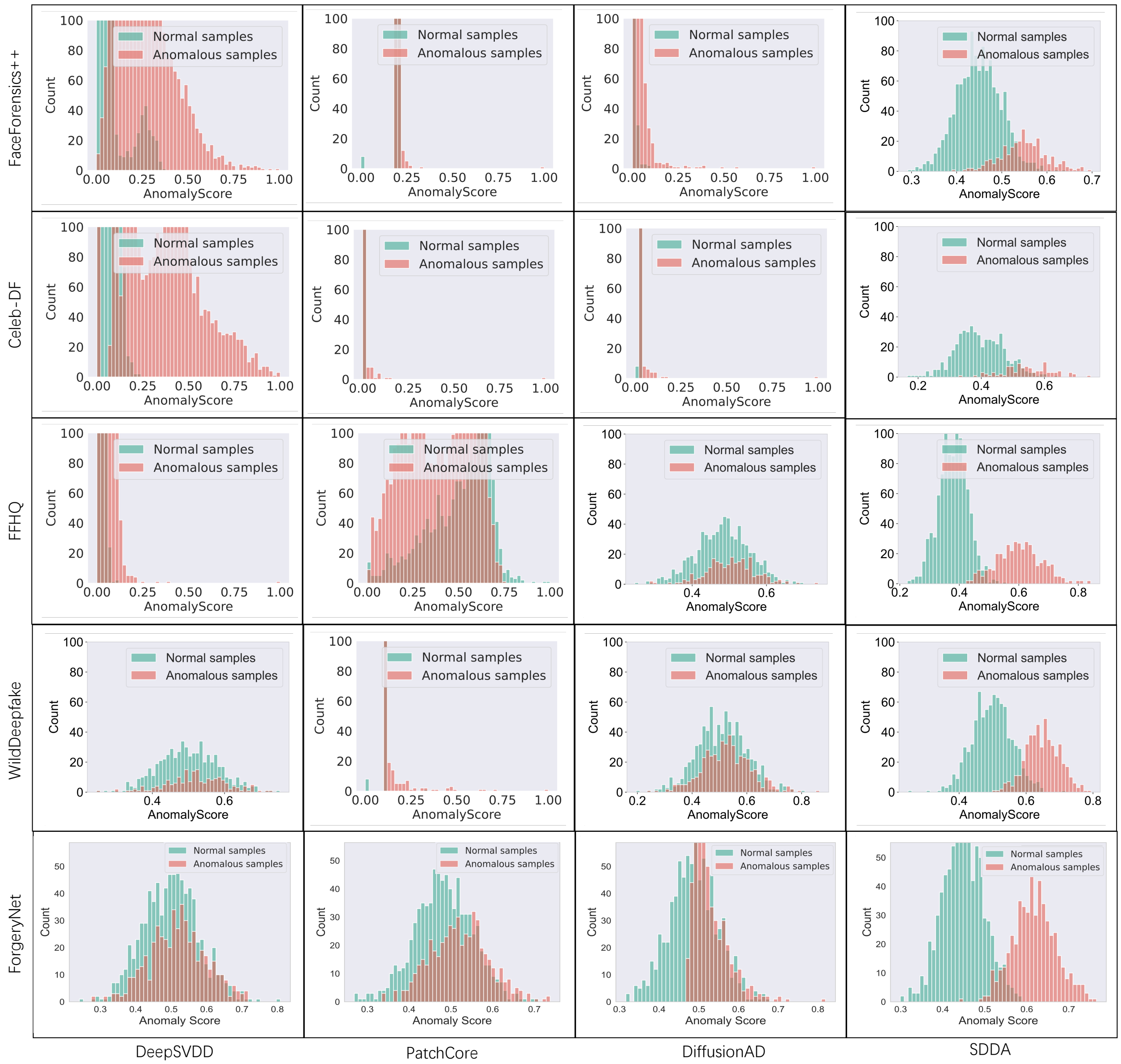

Figure 2: Anomaly scores visualization of SDDA and compared methods (DeepSVDD, PatchCore, and DiffusionAD) across five datasets: FaceForensics++, Celeb-DF, FFHQ, WildDeepfake, and ForgeryNet. Green bars represent real (normal) samples, and red bars represent fake (abnormal) samples. SDDA produces the most compact and separable clusters across all datasets, highlighting its superior dual-domain representation alignment and anomaly discriminability.

classification approaches, and feature distribution modeling techniques. We also highlight their common strengths, such as improved generalization to unseen manipulation methods, as well as their limitations, including sensitivity to domain shifts and difficulty in capturing subtle forgery artifacts.

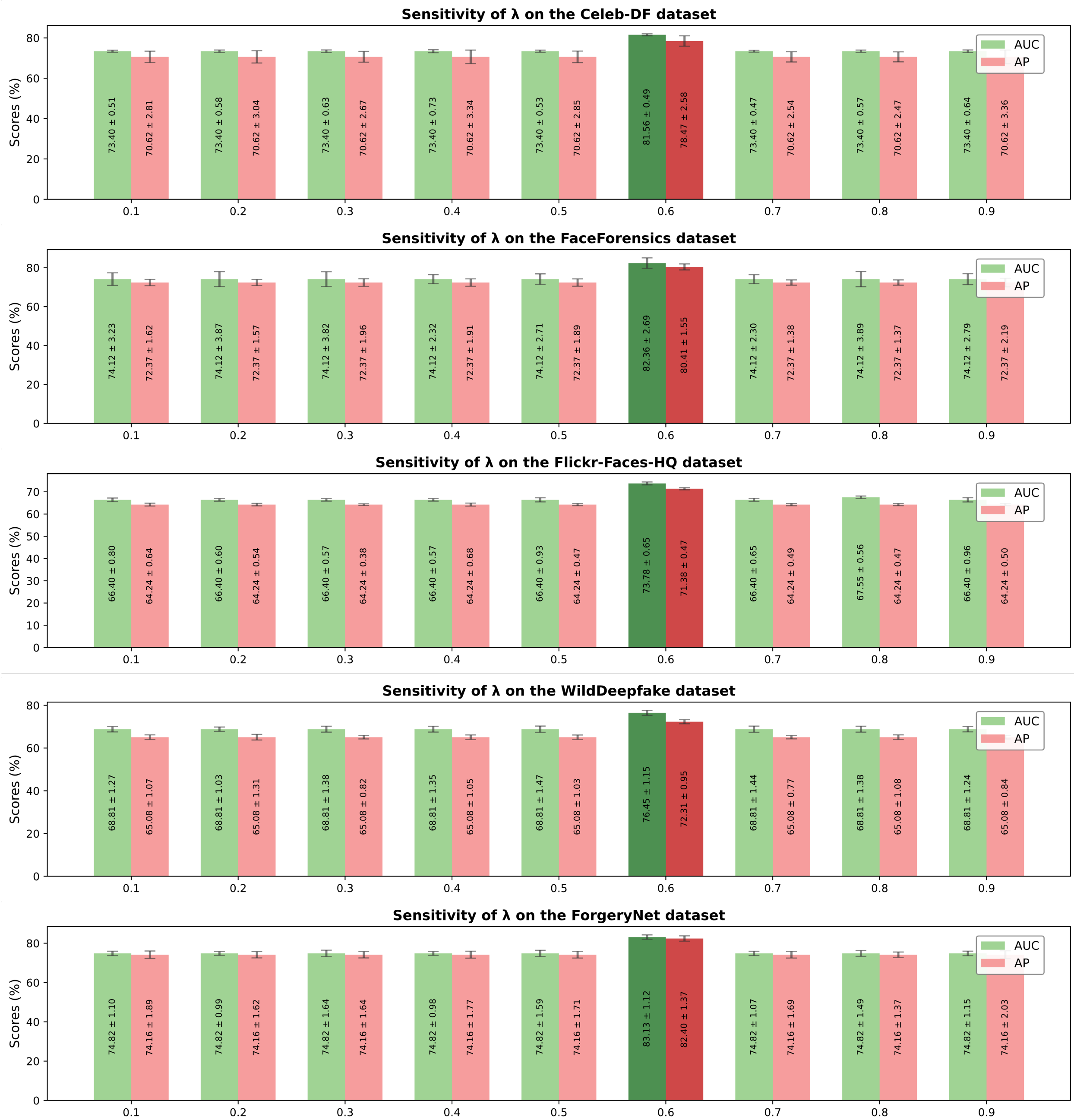

Figure 3: Parameter sensitivity analysis of SDDA on the five datasets: FaceForensics, Celeb-DF, Flickr-Faces-HQ, WildDeepfake, and ForgeryNet.

This addition provides a clearer context for our work and better positions our method within the broader landscape of anomaly detection–based deepfake detection research.

The specific revisions are shown below.

2.3 Anomaly Detection-Based Deepfake Detection Methods

Anomaly detection has become an important and rapidly developing research direction in the field of deepfakes in recent years, especially in addressing the problem of insufficient generalization ability of traditional supervised classification methods in open set scenarios, which has shown significant advantages.

Reconstruction-based methods train reconstruction models (such as autoencoders) using only real faces, allowing them to learn the distribution of real data. During testing, real images are typically reconstructed accurately, while forged images, due to containing aberrant tampering traces, often produce significant reconstruction errors. Deep forgery detection can be achieved by analyzing the differences between the original and reconstructed images. These methods do not require a large number of forged samples and have good generalization ability against unknown forgery methods. Shi et al. proposed FRG2D, an end-to-end face reconstruction-based framework for generalized deepfake detection. The method learns the distribution of authentic faces through reconstruction and identifies manipulated images by analyzing reconstruction discrepancies. To enhance feature extraction, FRG2D incorporates CBAM, CAB, and a Residual Outlook Attention (ROA) module. Experimental results demonstrate that FRG2D achieves strong generalization performance across unseen deepfake domains.

One-class classification methods are trained exclusively on authentic facial images to learn the feature distribution or decision boundary of real data. During inference, samples that deviate from this learned distribution are regarded as anomalies and are therefore classified as deepfakes. Since these methods do not rely on manipulated samples for supervised training, they generally exhibit strong generalization ability to unseen forgery methods. Khalid et al. proposed OC-FakeDect, a one-class deepfake detection method that trains a Variational Autoencoder (VAE) solely on authentic facial images and identifies manipulated images as anomalies, enabling effective generalization to unseen forgery methods. Cao et al. proposed OC-SAN, a one-class style autoencoder network for deepfake detection. The model learns identity and personalized style representations from authentic facial images and reconstructs only real faces effectively. Manipulated images are identified based on abnormal reconstruction patterns, enabling strong generalization to unseen forgery methods without requiring fake samples during training.

Feature distribution modeling methods learn the statistical distribution of authentic facial features and classify samples that deviate significantly from this distribution as deepfakes. By modeling the global structure of real facial representations, these methods generally exhibit strong generalization to unseen forgery methods. Khodabakhsh et al. proposed a two-stage anomaly detection method that learns the distribution of pristine facial images and detects manipulated images based on deviations from the estimated pixel-wise probabilities, achieving strong generalization to unseen synthesis methods. Maiano et al. proposed two out-of-distribution (OOD) detection methods for generalized deepfake detection. The first method detects manipulated images through reconstruction errors, while the second incorporates an attention mechanism to enhance anomaly localization. Both methods are designed to identify samples that deviate from the distribution of authentic images, demonstrating strong robustness to unseen deepfake generation methods.

Reviewer Comment 4: Method novelty

Reply: We thank the reviewer for this helpful suggestion. The specific revisions are shown below.

3 Methodology

3.9 Design Rationale and Key Innovations Scoring

To clarify the novelty of our framework, SDDA is not a simple integration of existing components.

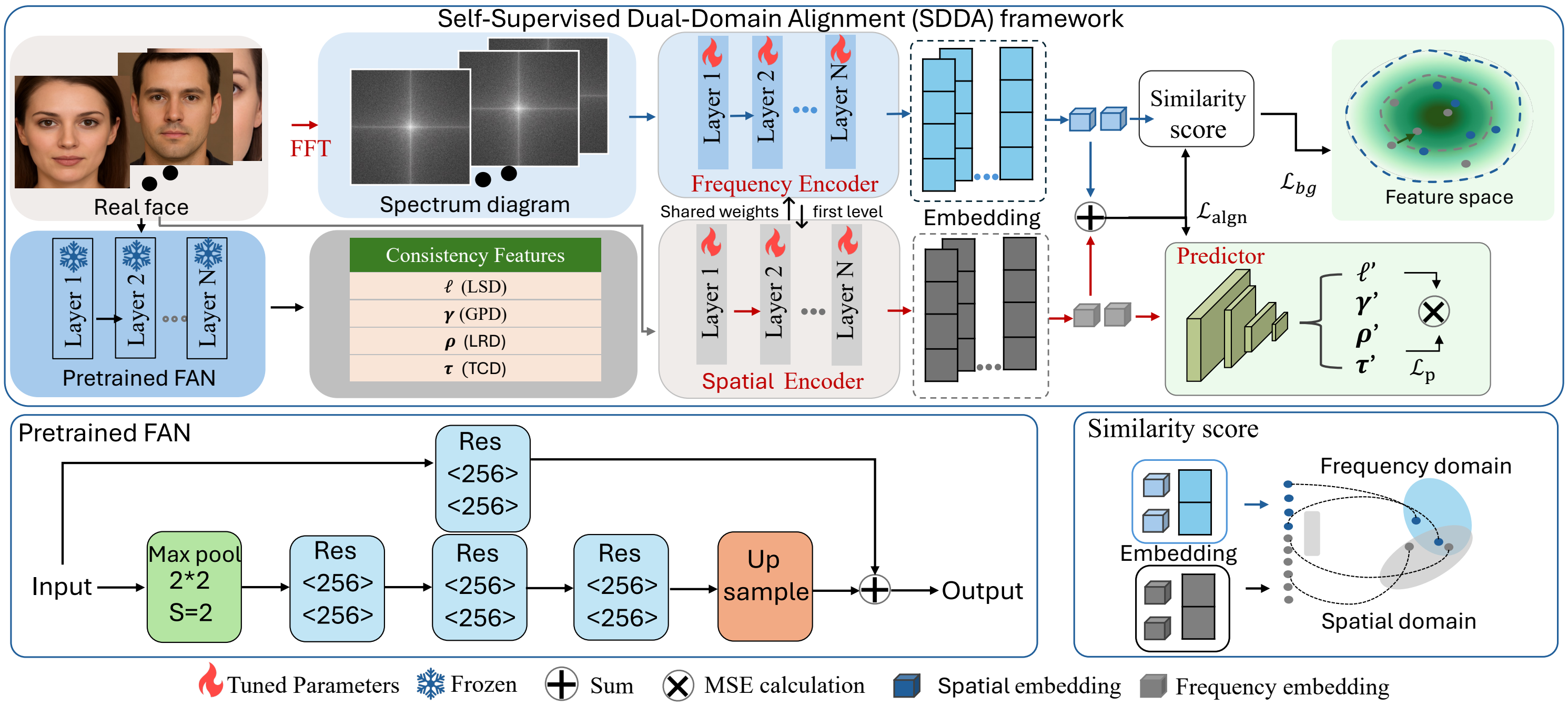

Figure 4: Overview of the proposed Self-Supervised Dual-Domain Alignment (SDDA) framework for deepfake anomaly detection.

Instead, it establishes a unified dual-domain self-supervised learning paradigm, where spatial and frequency representations are explicitly aligned through a consistency constraint rather than directly fused. In addition, a dual-domain contrastive objective is designed to enforce compactness of real samples while implicitly separating manipulated ones as out-of-distribution deviations. Finally, a hypersphere constraint is applied on the aligned representation space to further enhance intra-class compactness and inter-class separability. These designs jointly enable SDDA to effectively capture subtle forgery traces across both domains.

Reviewer Comment 5: Figure captions

Some figure captions are excessively long (in some cases exceeding ten lines). For better readability, the captions could be shortened while keeping only the essential information.

Reply: As requested by the reviewers, we revised some of the numerical headings and added the subsequent detailed descriptions to the main text. The specific revisions are shown below.

3 Methodology

...

The architecture contains three core components: (1) a shared-weight spatial–frequency encoder that learns domain-aligned representations through joint feature extraction; (2) a multi-objective feature-prediction head that estimates the four consistency descriptors ($\boldsymbol{\ell}$, $\boldsymbol{\gamma}$, $\boldsymbol{\rho}$, $\boldsymbol{\tau}$), enforcing fine-grained spatial–frequency correspondence and enhancing cross-domain discriminability; (3) a global–local anomaly scoring module that integrates multi-scale reconstruction with attention-guided deviation estimation to capture both structural and spectral abnormalities. By explicitly aligning dual-domain embeddings and constraining their consistency

through self-supervision, the framework establishes a theoretically grounded representation space that generalizes effectively to unseen manipulation types.

Reviewer Comment 6: LaTeX formatting

Reply: Thank you for pointing out this formatting issue. We sincerely appreciate the reviewer's careful reading and helpful suggestion. We have carefully proofread the manuscript and corrected the LaTeX quotation formatting (i.e., using “ and ” for paired quotes) throughout the revised version. These corrections help improve the overall clarity and presentation quality of the paper.

Reviewer Comment 7: Method description clarity

Some parts of the methodology would benefit from clearer explanations. In particular, the four self-supervised descriptors (LSD, GPD, LRD, and TCD) are introduced conceptually, but their exact computation and implementation details are not sufficiently described. Providing clearer definitions or additional explanations would improve reproducibility.

Reply: Thank you for pointing this out. We agree that the previous version only introduced LSD, GPD, LRD, and TCD at a conceptual level, which may limit reproducibility. In the revised manuscript, we have added explicit mathematical definitions and implementation details for the four self-supervised descriptors. Specifically, we now describe how LSD is computed from local gradient differences, how GPD is obtained from global feature correlation, how LRD is calculated from residual feature responses, and how TCD aggregates multi-layer feature deviations. We also clarify how these descriptors are concatenated into the self-supervised target vector and optimized through the descriptor prediction loss. The specific revisions are shown below.

Abstract

Deepfake anomaly detection has become increasingly critical in visual security applications. However, many existing methods rely heavily on large-scale supervised forgery annotations and often struggle to capture subtle manipulation traces distributed across both spatial and frequency domains. To address these limitations, we propose a Self-Supervised Dual-Domain Alignment (SDDA) framework that jointly learns spatial and spectral representations without requiring labeled forged samples. Specifically, SDDA employs two parameter-sharing encoders to extract complementary features from RGB images and their frequency-domain counterparts, while a cross-domain alignment module enforces consistency between spatial textures and spectral signatures. To provide explicit and reproducible self-supervised supervision, four complementary descriptors are further derived from FAN feature maps: Local Structural Deviation (LSD) for local gradient inconsistency, Global Pattern Discrepancy (GPD) for holistic channel-correlation differences, Local Residual Difference (LRD) for residual-level inconsistency, and Total Consistency Deviation (TCD) for multi-layer feature deviation. These descriptors are predicted from the fused spatial–frequency embedding, encouraging the model to learn manipulation-sensitive representations in the absence of fake samples. In addition, a dual-domain contrastive objective enhances the discrimination of subtle forgery-related anomalies while improving robustness against real-world degradations such as compression and illumination variations. Experimental results demonstrate that SDDA consistently outperforms state-of-the-art baselines, particularly under challenging unseen-manipulation scenarios. Overall, the proposed framework provides an interpretable and label-efficient solution for dual-domain deepfake anomaly detection.

3 Methodology

3.4 Self-supervised Descriptor Prediction Module

The four self-supervised descriptors are originally defined on the paired spatial and frequency representations I_s and I_f, as summarized in Table ??. To keep the formulation consistent with the original image-level definition, we do not redefine these descriptors at the feature level. Instead, we instantiate I_s and I_f with the response maps extracted from different layers of the two encoders during implementation.

Given the feature maps $\mathbf{F}_s^{(\ell)}$ and $\mathbf{F}_f^{(\ell)}$ from the ℓ-th layer of the spatial and frequency encoders, we first obtain their channel-averaged response maps:

$$I_s^{(\ell)} = \frac{1}{C_\ell}\sum_{c=1}^{C_\ell}\mathbf{F}_{s,c}^{(\ell)}, \quad I_f^{(\ell)} = \frac{1}{C_\ell}\sum_{c=1}^{C_\ell}\mathbf{F}_{f,c}^{(\ell)}. \tag{1}$$

Here, $I_s^{(\ell)}$ and $I_f^{(\ell)}$ are regarded as layer-wise instantiations of the original spatial and frequency representations I_s and I_f. Therefore, the following descriptors preserve the same mathematical meaning as the image-level definitions, while allowing them to be computed from the selected encoder layers.

Local Structural Deviation. Local Structural Deviation (LSD) measures local structural inconsistency between the spatial and frequency representations. Following the original definition, LSD is computed as the average gradient discrepancy:

$$y_{\mathrm{LSD}} = \frac{1}{N_2}\sum_{i=1}^{N_2}\left|\nabla I_s^{(2)}(i) - \nabla I_f^{(2)}(i)\right|^2, \tag{2}$$

where $I_s^{(2)}$ and $I_f^{(2)}$ denote the response maps extracted from the second encoder layer, and N_2 is the number of spatial positions in the response map. The gradient operator ∇ is implemented by local horizontal and vertical gradient filters. This descriptor encourages the model to preserve fine-grained local structural differences between the two domains.

Global Pattern Discrepancy. Global Pattern Discrepancy (GPD) captures the global distribution difference between the spatial and frequency representations. Consistent with the original image-level formulation, GPD is computed as the discrepancy between the mean responses of the two domains:

$$y_{\mathrm{GPD}} = \left\|\frac{1}{N_3}\sum_{i=1}^{N_3} I_s^{(3)}(i) - \frac{1}{N_3}\sum_{i=1}^{N_3} I_f^{(3)}(i)\right\|_2^2, \tag{3}$$

where $I_s^{(3)}$ and $I_f^{(3)}$ are obtained from the third encoder layer. Compared with LSD, GPD focuses more on holistic response distribution and global cross-domain consistency.

Local Residual Difference. Local Residual Difference (LRD) measures residual-level inconsistency between the spatial and frequency representations. Following the original definition, we first obtain the locally smoothed responses:

$$\hat{I}_s^{(3)} = \mathrm{AvgPool}_r(I_s^{(3)}), \quad \hat{I}_f^{(3)} = \mathrm{AvgPool}_r(I_f^{(3)}), \tag{4}$$

where $\mathrm{AvgPool}_r(\cdot)$ denotes average pooling with an $r \times r$ kernel. The LRD descriptor is then computed as:

$$y_{\mathrm{LRD}} = \frac{1}{N_3}\sum_{i=1}^{N_3}\left|\left(I_s^{(3)}(i) - \hat{I}_s^{(3)}(i)\right) - \left(I_f^{(3)}(i) - \hat{I}_f^{(3)}(i)\right)\right|. \tag{5}$$

This descriptor emphasizes local residual differences and is useful for capturing subtle manipulation traces that may be weakened in global representations.

Total Consistency Deviation. Total Consistency Deviation (TCD) measures the consistency deviation between deeper spatial and frequency representations. In accordance with the original definition, TCD is computed over multiple encoder layers. In our implementation, we use the second, third and fourth layers, denoted as $\mathcal{L} = \{2, 3, 4\}$. For each layer, the feature representation is obtained by global average pooling:

$$\phi_s^{(\ell)} = \mathrm{GAP}(\mathbf{F}_s^{(\ell)}), \quad \phi_f^{(\ell)} = \mathrm{GAP}(\mathbf{F}_f^{(\ell)}). \tag{6}$$

The TCD descriptor is formulated as:

$$y_{\mathrm{TCD}} = \frac{1}{|\mathcal{L}|} \sum_{\ell \in \mathcal{L}} \frac{\left\| \phi_s^{(\ell)} - \phi_f^{(\ell)} \right\|_2^2}{C_\ell}. \tag{7}$$

This descriptor provides a multi-level consistency constraint by aggregating deviations from different semantic layers.

The four descriptors are then concatenated to form the self-supervised target vector:

$$\mathbf{y} = [y_{\mathrm{LSD}}, y_{\mathrm{GPD}}, y_{\mathrm{LRD}}, y_{\mathrm{TCD}}] \in \mathbb{R}^4. \tag{8}$$

A prediction head $P_\phi(\cdot)$ takes the concatenated spatial–frequency embedding as input:

$$\mathbf{z} = [\mathbf{z}_s \| \mathbf{z}_f], \tag{9}$$

and predicts the descriptor vector:

$$\hat{\mathbf{y}} = P_\phi(\mathbf{z}). \tag{10}$$

The descriptor prediction loss is formulated as:

$$\mathcal{L}_p = \frac{1}{N} \sum_{i=1}^{N} \sum_{m \in \mathcal{M}} \lambda_m \left\| \hat{y}_m^{(i)} - y_m^{(i)} \right\|_2^2, \tag{11}$$

where $\mathcal{M} = \{\mathrm{LSD}, \mathrm{GPD}, \mathrm{LRD}, \mathrm{TCD}\}$, and λ_m denotes the loss weight of each descriptor. In our implementation, all λ_m are set to 1 unless otherwise specified. To avoid scale imbalance among different descriptors, each descriptor is normalized before loss computation.
