# Supplementary material for: Dual-domain self-supervised feature alignment via spectral–spatial representation learning for deepfake anomaly detection
Source: PLoS One. 2026 Sep 11;21(9):e0358049. doi: 10.1371/journal.pone.0358049 (PMC13568503; doi:10.1371/journal.pone.0358049)
Supplement: Peer review history

# Peer review: Decision Letter 0

**DOI:** 10.1371/journal.pone.0358049.r001

**Authors:** Feng Ding

5 Jan 2026

Dear Dr. He,

Thank you for submitting your manuscript to PLOS ONE. After careful consideration, we feel that it has merit but does not fully meet PLOS ONE’s publication criteria as it currently stands. Therefore, we invite you to submit a revised version of the manuscript that addresses the points raised during the review process.

Please submit your revised manuscript by Feb 19 2026 11:59PM. If you will need more time than this to complete your revisions, please reply to this message or contact the journal office at plosone@plos.org. When you're ready to submit your revision, log on to https://www.editorialmanager.com/pone/ and select the 'Submissions Needing Revision' folder to locate your manuscript file.

- A letter that responds to each point raised by the academic editor and reviewer(s). You should upload this letter as a separate file labeled 'Response to Reviewers'.
- A marked-up copy of your manuscript that highlights changes made to the original version. You should upload this as a separate file labeled 'Revised Manuscript with Track Changes'.
- An unmarked version of your revised paper without tracked changes. You should upload this as a separate file labeled 'Manuscript'.

If you would like to make changes to your financial disclosure, please include your updated statement in your cover letter. Guidelines for resubmitting your figure files are available below the reviewer comments at the end of this letter.

If applicable, we recommend that you deposit your laboratory protocols in protocols.io to enhance the reproducibility of your results. Protocols.io assigns your protocol its own identifier (DOI) so that it can be cited independently in the future. For instructions see: https://journals.plos.org/plosone/s/submission-guidelines#loc-laboratory-protocols. Additionally, PLOS ONE offers an option for publishing peer-reviewed Lab Protocol articles, which describe protocols hosted on protocols.io. Read more information on sharing protocols at https://plos.org/protocols?utm_medium=editorial-email&utm_source=authorletters&utm_campaign=protocols.

We look forward to receiving your revised manuscript.

Kind regards,

Feng Ding

Academic Editor

PLOS One

Journal Requirements:

When submitting your revision, we need you to address these additional requirements.

1. Please ensure that your manuscript meets PLOS ONE's style requirements, including those for file naming. The PLOS ONE style templates can be found at

https://journals.plos.org/plosone/s/file?id=wjVg/PLOSOne_formatting_sample_main_body.pdf and

https://journals.plos.org/plosone/s/file?id=ba62/PLOSOne_formatting_sample_title_authors_affiliations.pdf

2. Please note that PLOS One has specific guidelines on code sharing for submissions in which author-generated code underpins the findings in the manuscript. In these cases, we expect all author-generated code to be made available without restrictions upon publication of the work. Please review our guidelines at https://journals.plos.org/plosone/s/materials-and-software-sharing#loc-sharing-code and ensure that your code is shared in a way that follows best practice and facilitates reproducibility and reuse.

3. We note that your Data Availability Statement is currently as follows: All relevant data are within the manuscript and its Supporting Information files.

Please confirm at this time whether or not your submission contains all raw data required to replicate the results of your study. Authors must share the “minimal data set” for their submission. PLOS defines the minimal data set to consist of the data required to replicate all study findings reported in the article, as well as related metadata and methods (https://journals.plos.org/plosone/s/data-availability#loc-minimal-data-set-definition).

For example, authors should submit the following data:

- The values behind the means, standard deviations and other measures reported;

- The values used to build graphs;

- The points extracted from images for analysis.

Authors do not need to submit their entire data set if only a portion of the data was used in the reported study.

If your submission does not contain these data, please either upload them as Supporting Information files or deposit them to a stable, public repository and provide us with the relevant URLs, DOIs, or accession numbers. For a list of recommended repositories, please see https://journals.plos.org/plosone/s/recommended-repositories.

If there are ethical or legal restrictions on sharing a de-identified data set, please explain them in detail (e.g., data contain potentially sensitive information, data are owned by a third-party organization, etc.) and who has imposed them (e.g., an ethics committee). Please also provide contact information for a data access committee, ethics committee, or other institutional body to which data requests may be sent. If data are owned by a third party, please indicate how others may request data access.

4. PLOS requires an ORCID iD for the corresponding author in Editorial Manager on papers submitted after December 6th, 2016. Please ensure that you have an ORCID iD and that it is validated in Editorial Manager. To do this, go to ‘Update my Information’ (in the upper left-hand corner of the main menu), and click on the Fetch/Validate link next to the ORCID field. This will take you to the ORCID site and allow you to create a new iD or authenticate a pre-existing iD in Editorial Manager.

5. We note that Figure 1 and 2 includes an image of a participant.

As per the PLOS ONE policy (http://journals.plos.org/plosone/s/submission-guidelines#loc-human-subjects-research) on papers that include identifying, or potentially identifying, information, the individual(s) or parent(s)/guardian(s) must be informed of the terms of the PLOS open-access (CC-BY) license and provide specific permission for publication of these details under the terms of this license. Please download the Consent Form for Publication in a PLOS Journal (http://journals.plos.org/plosone/s/file?id=8ce6/plos-consent-form-english.pdf). The signed consent form should not be submitted with the manuscript, but should be securely filed in the individual's case notes. Please amend the methods section and ethics statement of the manuscript to explicitly state that the patient/participant has provided consent for publication: “The individual in this manuscript has given written informed consent (as outlined in PLOS consent form) to publish these case details”.

Please respond by return e-mail with an amended manuscript. We can upload this to your submission on your behalf.

If you are unable to obtain consent from the subject of the photograph, please either instruct us to remove the figure or supply a replacement figure by return e-mail for which you hold the relevant copyright permissions and subject consents. In some cases, you may need to specify in the text that the image used in the figure is not the original image used in the study, but a similar image used for illustrative purposes only. We can make any changes on your behalf.

6. We note you have included a table to which you do not refer in the text of your manuscript. Please ensure that you refer to Table 2 - 8 in your text; if accepted, production will need this reference to link the reader to the Table.

7. If the reviewer comments include a recommendation to cite specific previously published works, please review and evaluate these publications to determine whether they are relevant and should be cited. There is no requirement to cite these works unless the editor has indicated otherwise.

[Note: HTML markup is below. Please do not edit.]

Reviewers' comments:

Reviewer's Responses to Questions

**Comments to the Author**

1. Is the manuscript technically sound, and do the data support the conclusions?

Reviewer #1: Yes

Reviewer #2: Yes

Reviewer #3: Yes

**********

2. Has the statistical analysis been performed appropriately and rigorously?

Reviewer #1: I Don't Know

Reviewer #2: Yes

Reviewer #3: Yes

**********

3. Have the authors made all data underlying the findings in their manuscript fully available?

The PLOS Data policy

Reviewer #1: Yes

Reviewer #2: Yes

Reviewer #3: Yes

**********

4. Is the manuscript presented in an intelligible fashion and written in standard English?

Reviewer #1: Yes

Reviewer #2: Yes

Reviewer #3: Yes

**********

Reviewer #1: This paper proposes SDDA, a self-supervised dual-domain alignment framework for deepfake anomaly detection without using forged samples in training. It learns shared spatial–frequency representations via multi-feature prediction and domain alignment, then scores test samples by deviation from a learned hypersphere center. Experiments on multiple benchmarks compare SDDA with representative anomaly-detection baselines and report improved cross-dataset generalization.

To further improve the quality of this paper, I advise the authors to incorporate the following comments.

1.Several baseline names are written inconsistently (e.g., CFlow vs. CFLow, AECNN vs. AE-CNN). Please standardize the naming throughout the manuscript for clarity.

2. In Introduction section, it lists FF++, CelebDF, DFDC, and DeeperForensics as the experimental datasets, whereas the Experiments section actually uses FF++, CelebDF, FFHQ, and WildDeepfake. Dataset description is inconsistent between sections.

3. The manuscript contains multiple placeholders (e.g., “Table ??”) and even “placeholder SA” in the references. These must be removed before resubmission.

4. The symbols Is, If, Is, If are not clearly defined in the main text. Please explicitly specify whether they refer to the raw images, frequency spectra, or FAN feature maps.

5. FFHQ is introduced as a dataset containing only real images, yet the experimental setup states that the validation and test sets are balanced mixtures of real and fake samples. Since FFHQ does not provide a native fake subset, the source and construction of the fake samples must be clearly described.

6. The contributions emphasize a “unified anomaly scoring mechanism” combining global reconstruction errors with local frequency-domain prediction discrepancies. However, Section 3.8 defines the anomaly score solely as the sum of distances to the center c (Eq. 11), with no reconstruction branch or score fusion explicitly specified.

7. The preprocessing applies a 2D DFT to RGB inputs, but Table 4 describes the encoder as a four-layer Conv1D network. The paper should explain how 2D images/spectra are converted into a 1D sequence input (e.g., flattening strategy, tensor dimensions, and whether channels are processed separately).

8. The method name shown at the bottom of Fig. 3 is “DFAD,” which is inconsistent with the main text. Please correct the figure labeling to match the manuscript.

9. Some related works should be discussed, such as 10.1109/TCSVT.2022.3204753, 10.1109/TCSVT.2023.3278310, 10.1109/TCSVT.2024.3510355, 10.1109/TMM.2024.3415415

Reviewer #2: This manuscript introduces SDDA, a novel self-supervised dual-domain feature alignment framework for deepfake anomaly detection. The authors propose learning spatial and spectral representations jointly, using shared-weight encoders, multi-feature prediction, and hypersphere compactness to improve generalization and robustness. The method is evaluated on multiple public datasets and compared to a range of state-of-the-art baselines. The manuscript presents an interesting contribution to the field of deepfake anomaly detection, pending minor revisions as suggested below.

1. Reproducibility and Implementation Details:

While the high-level description of the framework is clear, some implementation details are lacking. For example, more specifics on data preprocessing, exact hyperparameters, and network architecture (such as layer counts, activation functions, and regularization strategies) would improve reproducibility.

2. Comparison to Recent Related Work:

Although a wide array of baselines is included, some very recent works in self-supervised or frequency-based deepfake detection may not have been considered. The authors should clarify the selection criteria for baselines and discuss how their approach compares to the latest transformer-based or hybrid anomaly detectors.

3. Analysis of Failure Cases:

The manuscript would benefit from a qualitative analysis of failure cases or limitations—e.g., scenarios where SDDA underperforms, or types of deepfake manipulations that remain challenging.

4. Ablation Study Clarity:

The ablation table labels (wo-F, wo-L, etc.) could be more clearly linked to the modules they ablate, perhaps by including a short description in the table caption or main text.

5. Typographical Errors:

Some minor typographical errors and formatting inconsistencies (e.g., missing table numbers or question marks) should be corrected prior to publication.

Reviewer #3: This paper proposes a novel Self-Supervised Dual-Domain Alignment (SDDA) framework that jointly learns spatial and spectral representations without requiring labeled forgeries. This results in a significant gap in the models' ability to interpret and understand deepfake.

In brief, I agree with the innovations proposed in this paper, and the quality of the article basically meets the requirements of the journal PLOS ONE. However, there are still some issues that need to be addressed.

The paper suffer from the following major flaws:

1.Some modules in Figure 1 are overly simplified and roughly represented. For example, the "Time" module in the description of SDDA lacks precision. It is recommended to change it to "spatial features" or "spatial representation."

2.Extracting frequency-domain features as a complement to RGB features is a common approach in the field of deepfake detection. However, the motivation for selecting FFT in this work is not clearly justified. It is recommended to add ablation experiments comparing FFT with other methods for extracting frequency-domain features.

3.From the results in Table 5 and Table 6, it can be seen that the method proposed in this paper achieves superior performance. However, to better highlight the novelty and contribution of this work, it is recommended to incorporate comparisons with several relevant studies published in 2025.

4.Typos：

(1)The table numbering is incorrect. For example, page 11 line 312, page 14 line 357

(2)The last column in Figure 3 should represent this paper's method, SDDA. Could you please clarify why it is labeled as "DFAD" instead?

**********

what does this mean?). If published, this will include your full peer review and any attached files.

If you choose “no”, your identity will remain anonymous but your review may still be made public.

**Do you want your identity to be public for this peer review?**  For information about this choice, including consent withdrawal, please see our Privacy Policy

Reviewer #1: No

Reviewer #2: No

Reviewer #3: No

**********

[NOTE: If reviewer comments were submitted as an attachment file, they will be attached to this email and accessible via the submission site. Please log into your account, locate the manuscript record, and check for the action link "View Attachments". If this link does not appear, there are no attachment files.]

To ensure your figures meet our technical requirements, please review our figure guidelines: https://journals.plos.org/plosone/s/figures

You may also use PLOS’s free figure tool, NAAS, to help you prepare publication quality figures: https://journals.plos.org/plosone/s/figures#loc-tools-for-figure-preparation.

NAAS will assess whether your figures meet our technical requirements by comparing each figure against our figure specifications.

# Author comment: Author response to Decision Letter 1

**DOI:** 10.1371/journal.pone.0358049.r002

19 Jan 2026

We sincerely thank the Academic Editor for the careful handling of our manuscript and for the constructive suggestions. In response to the editor’s comments, we have thoroughly revised the manuscript to en-sure full compliance with PLOS One formatting and submission requirements. Specifically, we improved the clarity of the method description, standardized terminology across sections, corrected figure and table references, and refined the dataset descriptions. All modifications to the manuscript are explicitly marked in blue in the revised version.

Attachment Submitted filename: Response to Reviewers.pdf

# Peer review: Decision Letter 1

**DOI:** 10.1371/journal.pone.0358049.r003

**Authors:** Feng Ding

20 Apr 2026

Dear Dr. He,

Thank you for submitting your manuscript to PLOS ONE. After careful consideration, we feel that it has merit but does not fully meet PLOS ONE’s publication criteria as it currently stands. Therefore, we invite you to submit a revised version of the manuscript that addresses the points raised during the review process.

Please submit your revised manuscript by Jun 04 2026 11:59PM. If you will need more time than this to complete your revisions, please reply to this message or contact the journal office at plosone@plos.org. When you're ready to submit your revision, log on to https://www.editorialmanager.com/pone/ and select the 'Submissions Needing Revision' folder to locate your manuscript file.

- A letter that responds to each point raised by the academic editor and reviewer(s). You should upload this letter as a separate file labeled 'Response to Reviewers'.
- A marked-up copy of your manuscript that highlights changes made to the original version. You should upload this as a separate file labeled 'Revised Manuscript with Track Changes'.
- An unmarked version of your revised paper without tracked changes. You should upload this as a separate file labeled 'Manuscript'.

If you would like to make changes to your financial disclosure, please include your updated statement in your cover letter. Guidelines for resubmitting your figure files are available below the reviewer comments at the end of this letter.

If applicable, we recommend that you deposit your laboratory protocols in protocols.io to enhance the reproducibility of your results. Protocols.io assigns your protocol its own identifier (DOI) so that it can be cited independently in the future. For instructions see: https://journals.plos.org/plosone/s/submission-guidelines#loc-laboratory-protocols. Additionally, PLOS ONE offers an option for publishing peer-reviewed Lab Protocol articles, which describe protocols hosted on protocols.io. Read more information on sharing protocols at https://plos.org/protocols?utm_medium=editorial-email&utm_source=authorletters&utm_campaign=protocols.

As the corresponding author, your ORCID iD is verified in the submission system and will appear in the published article. PLOS supports the use of ORCID, and we encourage all coauthors to register for an ORCID iD and use it as well. Please encourage your coauthors to verify their ORCID iD within the submission system before final acceptance, as unverified ORCID iDs will not appear in the published article. *Only*  the individual author can complete the verification step; PLOS staff *cannot*  verify ORCID iDs on behalf of authors.

We look forward to receiving your revised manuscript.

Kind regards,

Feng Ding

Academic Editor

PLOS One

Journal Requirements:

1. If the reviewer comments include a recommendation to cite specific previously published works, please review and evaluate these publications to determine whether they are relevant and should be cited. There is no requirement to cite these works unless the editor has indicated otherwise.

2. Please review your reference list to ensure that it is complete and correct. If you have cited papers that have been retracted, please include the rationale for doing so in the manuscript text, or remove these references and replace them with relevant current references. Any changes to the reference list should be mentioned in the rebuttal letter that accompanies your revised manuscript. If you need to cite a retracted article, indicate the article’s retracted status in the References list and also include a citation and full reference for the retraction notice.

[Note: HTML markup is below. Please do not edit.]

Reviewers' comments:

Reviewer's Responses to Questions

**Comments to the Author**

Reviewer #1: (No Response)

Reviewer #4: (No Response)

**********

2. Is the manuscript technically sound, and do the data support the conclusions?

Reviewer #1: (No Response)

Reviewer #4: No

**********

3. Has the statistical analysis been performed appropriately and rigorously?

Reviewer #1: (No Response)

Reviewer #4: No

**********

4. Have the authors made all data underlying the findings in their manuscript fully available?

The PLOS Data policy

Reviewer #1: (No Response)

Reviewer #4: No

**********

5. Is the manuscript presented in an intelligible fashion and written in standard English?

Reviewer #1: (No Response)

Reviewer #4: Yes

**********

Reviewer #1: (No Response)

Reviewer #4: Overall, the paper presents an interesting attempt to integrate spatial and frequency-domain representations for deepfake anomaly detection. However, several methodological clarifications and experimental improvements are needed to better demonstrate the novelty and robustness of the proposed approach.

1 Dataset selection

The evaluation relies on four datasets: FaceForensics++, Celeb-DF, Flickr-Faces-HQ, and WildDeepfake. Most of these datasets were introduced before 2020. Considering the rapid progress in deepfake generation techniques, it would strengthen the paper to include evaluations on more recent datasets or provide justification for relying solely on older benchmarks.

2 Evaluation protocol

The training set contains only authentic samples, while both the validation and test sets include real and fake samples. However, it appears that the same manipulation methods are present in both validation and test sets. This may expose the model to the anomaly distribution during model selection. It would be helpful to clarify this setting and consider cross-manipulation evaluation to better demonstrate generalization to unseen forgeries.

3 Related work coverage

The related work section introduces only a limited number of anomaly detection–based deepfake detection approaches. Given the growing literature in this direction, the authors may consider adding a dedicated subsection summarizing existing anomaly detection methods to provide a more comprehensive overview.

4 Method novelty

The proposed framework combines several existing components, including spatial–frequency representations, feature prediction objectives, and hypersphere-based compactness constraints. While the integration is interesting, the paper would benefit from a clearer discussion of the specific novelty and advantages compared with prior work.

5 Figure captions

Some figure captions are excessively long (in some cases exceeding ten lines). For better readability, the captions could be shortened while keeping only the essential information.

6 LaTeX formatting

There are minor formatting issues in the manuscript. For example, paired quotation marks should be written as `` and '' in LaTeX. A careful proofreading of the manuscript would improve the overall presentation.

7 Method description clarity

Some parts of the methodology would benefit from clearer explanations. In particular, the four self-supervised descriptors (LSD, GPD, LRD, and TCD) are introduced conceptually, but their exact computation and implementation details are not sufficiently described. Providing clearer definitions or additional explanations would improve reproducibility.

**********

what does this mean?). If published, this will include your full peer review and any attached files.

If you choose “no”, your identity will remain anonymous but your review may still be made public.

**Do you want your identity to be public for this peer review?**  For information about this choice, including consent withdrawal, please see our Privacy Policy

Reviewer #1: No

Reviewer #4: No

**********

[NOTE: If reviewer comments were submitted as an attachment file, they will be attached to this email and accessible via the submission site. Please log into your account, locate the manuscript record, and check for the action link "View Attachments". If this link does not appear, there are no attachment files.]

To ensure your figures meet our technical requirements, please review our figure guidelines: https://journals.plos.org/plosone/s/figures

You may also use PLOS’s free figure tool, NAAS, to help you prepare publication quality figures: https://journals.plos.org/plosone/s/figures#loc-tools-for-figure-preparation.

NAAS will assess whether your figures meet our technical requirements by comparing each figure against our figure specifications.

# Author comment: Author response to Decision Letter 2

**DOI:** 10.1371/journal.pone.0358049.r004

16 May 2026

We sincerely thank the Academic Editor for the careful handling of our manuscript and for providing us with the opportunity to revise our work.

We have carefully addressed all comments from the reviewers and revised the manuscript accordingly. The revisions mainly focus on improving the experimental design, clarifying the methodology, enhancing reproducibility, and strengthening the overall presentation of the paper.

All changes have been clearly marked in the revised manuscript with track changes. Detailed point-by-point responses to the reviewers’ comments are provided in the accompanying “Response to Reviewers” document. We hope that the revised manuscript meets the journal’s requirements, and we appreciate your consideration.

Attachment Submitted filename: Response_to_Reviewers_auresp_2.pdf

# Peer review: Decision Letter 2

**DOI:** 10.1371/journal.pone.0358049.r005

**Authors:** Suo Gao

26 Aug 2026

Dual-Domain Self-Supervised Feature Alignment via Spectral–Spatial Representation Learning for Deepfake Anomaly Detection

PONE-D-25-63428R2

Dear Dr. He,

We’re pleased to inform you that your manuscript has been judged scientifically suitable for publication and will be formally accepted for publication once it meets all outstanding technical requirements.

Within one week, you’ll receive an e-mail detailing the required amendments. When these have been addressed, you’ll receive a formal acceptance letter and your manuscript will be scheduled for publication.

An invoice will be generated when your article is formally accepted. Please note, if your institution has a publishing partnership with PLOS and your article meets the relevant criteria, all or part of your publication costs will be covered. Please make sure your user information is up-to-date by logging into Editorial Manager at Editorial Manager® and clicking the ‘Update My Information' link at the top of the page. For questions related to billing, please contact billing support.

If your institution or institutions have a press office, please notify them about your upcoming paper to help maximize its impact. If they’ll be preparing press materials, please inform our press team as soon as possible -- no later than 48 hours after receiving the formal acceptance. Your manuscript will remain under strict press embargo until 2 pm Eastern Time on the date of publication. For more information, please contact onepress@plos.org.

Kind regards,

Suo Gao

Academic Editor

PLOS One

Additional Editor Comments (optional):

Reviewers' comments:

Reviewer's Responses to Questions

**Comments to the Author**

Reviewer #1: (No Response)

Reviewer #4: All comments have been addressed

**********

2. Is the manuscript technically sound, and do the data support the conclusions?

Reviewer #1: (No Response)

Reviewer #4: Yes

**********

3. Has the statistical analysis been performed appropriately and rigorously?

Reviewer #1: (No Response)

Reviewer #4: Yes

**********

4. Have the authors made all data underlying the findings in their manuscript fully available?

The PLOS Data policy

Reviewer #1: (No Response)

Reviewer #4: Yes

**********

5. Is the manuscript presented in an intelligible fashion and written in standard English?

Reviewer #1: (No Response)

Reviewer #4: Yes

**********

Reviewer #1: (No Response)

Reviewer #4: (No Response)

**********

what does this mean?). If published, this will include your full peer review and any attached files.

If you choose “no”, your identity will remain anonymous but your review may still be made public.

**Do you want your identity to be public for this peer review?**  For information about this choice, including consent withdrawal, please see our Privacy Policy

Reviewer #1: No

Reviewer #4: **Yes:** Kuiyuan Zhang

**********

# Editor report: Acceptance letter

**DOI:** 10.1371/journal.pone.0358049.r006

**Authors:** Suo Gao

PONE-D-25-63428R2

PLOS One

Dear Dr. He,

I'm pleased to inform you that your manuscript has been deemed suitable for publication in PLOS One. Congratulations! Your manuscript is now being handed over to our production team.

At this stage, our production department will prepare your paper for publication. This includes ensuring the following:

* All references, tables, and figures are properly cited

* All relevant supporting information is included in the manuscript submission,

* There are no issues that prevent the paper from being properly typeset

You will receive further instructions from the production team, including instructions on how to review your proof when it is ready. Please keep in mind that we are working through a large volume of accepted articles, so please give us a few days to review your paper and let you know the next and final steps.

Lastly, if your institution or institutions have a press office, please let them know about your upcoming paper now to help maximize its impact. If they'll be preparing press materials, please inform our press team within the next 48 hours. Your manuscript will remain under strict press embargo until 2 pm Eastern Time on the date of publication. For more information, please contact onepress@plos.org.

You will receive an invoice from PLOS for your publication fee after your manuscript has reached the completed accept phase. If you receive an email requesting payment before acceptance or for any other service, this may be a phishing scheme. Learn how to identify phishing emails and protect your accounts at https://explore.plos.org/phishing.

If we can help with anything else, please email us at customercare@plos.org.

Thank you for submitting your work to PLOS One and supporting open access.

Kind regards,

PLOS One Editorial Office Staff

on behalf of

Professor Suo Gao

Academic Editor

PLOS One
